# BAM-BepA complexes in outer membrane protein quality control

**DOI:** 10.1038/s41467-026-75227-x

**Published:** 2026-07-09

**Authors:** Katherine L. Fenn, Victoria Higgins, Jonathan M. Machin, Antonio N. Calabrese, Alan Berry, Sheena E. Radford, Neil A. Ranson

**Affiliations:** Astbury Centre for Structural Molecular Biology and School of Molecular and Cellular Biology, Faculty of Biological Sciences, University of Leeds, Leeds, UK

**Keywords:** Cryoelectron microscopy, Bacteriology, Enzyme mechanisms, Membrane structure and assembly

## Abstract

Correct folding of outer membrane proteins (OMPs) by the β-barrel assembly machinery (BAM) is essential for maintaining the outer membrane (OM) barrier function of diderm bacteria. When OMP biogenesis is perturbed, the β-barrel assembly enhancing protease A (BepA) binds to BAM to mediate quality control, but how BepA interacts with BAM and degrades substrate OMPs remains unclear. Here, cryoEM structures of BAM-bound BepA reveals that BepA induces large conformational changes in the BAM complex enabling the enzyme to poise its active site within the periplasmic ring of BAM, beneath the BamA barrel. The lid of BepA is dynamic, embedding two of its water-soluble helices deep into the membrane bilayer when BAM-bound, which readies BepA for proteolysis of misfolding OMPs. Movement of BepA’s plug is triggered by OMP binding rather than interaction with BAM, activating the enzyme for cleavage. We reveal BepA preferentially recognises Aromatic-X-Aromatic (Ar-X-Ar) motifs which are enriched in OMP sequences. The results reveal a mechanism for proteolytic degradation by BepA in OMP quality control which requires interaction with BAM, the membrane, and its OMP substrates.

## Abstract (web summary)

Here the authors show that BepA is activated to degrade stalled outer membrane proteins on BAM through binding to BAM, inducing conformational changes, membrane embedding its water-soluble lid, and triggering plug movement upon substrate binding to enable proteolysis.

## Introduction

The outer membrane (OM) of diderm bacteria acts as an essential barrier between the cell and its environment^1^. Densely packed throughout the OM are β-barrel outer membrane proteins (OMPs) that are integral to the OM’s architecture and perform diverse roles that include exchange of nutrients and waste products, export of virulence factors, and OM maintenance and assembly^2,3^. The correct and efficient assembly of OMPs is therefore essential for bacterial viability and pathogenesis^4^.

OMP biogenesis begins in the cytoplasm and culminates in nascent OMP folding and insertion into the OM. In *E. coli*, the OMP polypeptide chain is synthesised in the cytoplasm and transported across the inner membrane (IM) via the SEC machinery in an unfolded state. The periplasm-resident chaperone SurA binds newly translocated OMPs in the periplasm, maintaining them in an unfolded state and preventing their aggregation^5–10^. SurA also binds the β-barrel assembly machinery (BAM) at the OM and, together, BAM-SurA complexes mediate the transition of the OMP from an unfolded state into a folded β-barrel that is inserted in the OM bilayer^10–12^. *E. coli* BAM is a highly-conserved, multi-protein complex composed of the core component BamA and four accessory lipoproteins, BamB-E. BamA, itself an OMP, contains a 16-stranded C-terminal OM embedded β-barrel domain, and five N-terminal polypeptide transport-associated (POTRA) domains that extend into the periplasm (Supplementary Fig. 1a) and mediate interactions with the BamB-E lipoproteins, as well as SurA^11,12^. Structural studies have revealed the dynamic transitions of BAM required for OMP folding^11,13–15^ (recently reviewed by Birtles et al.^16^) and have shown that the first strand of the BamA barrel (β1) binds the C-terminal region of the incoming OMP (known as the β-signal), forming an anti-parallel intermolecular β-strand pairing. Subsequent β-strands template against this initial strand until a hybrid barrel is formed prior to the folded OMP’s release into the membrane^11–14,17–21^.

BAM can fold OMPs in just a few seconds^22^, but OMP folding is not error-free^23^. Misfolded or stalled OMPs pose a significant risk to cellular integrity, and the accumulation of unfolded OMPs in the periplasm can compromise OM barrier function^4^. To mitigate this risk, quality control mechanisms monitor and manage OMP biogenesis^24,25^. The sigmaE stress response is activated when unfolded OMPs accumulate in the periplasm^26^ and has a three-pronged effect on OMP biogenesis: to increase OMP folding capability, degrade misfolded OMPs and reduce the number of OMPs that require folding^4^. Accordingly, expression of the BAM complex subunits and SurA are upregulated by sigmaE to increase OMP folding, whilst expression of periplasmic chaperones and proteases such as Skp, DegP and BepA are also upregulated to coordinate the removal of misfolded/aggregated OMPs. Finally, OMP flux is reduced by small noncoding RNAs that repress the cytoplasmic expression of the most abundant OMPs, OmpC, OmpF and OmpA^4^.

The β-barrel assembly enhancing protease (BepA, formally known as YfgC) has been shown previously to interact with BAM^27^ and with OMP substrates folding on BAM^28^. BepA is thought to act when OMPs are stalled late in folding and has been described, therefore, as the last resort protease^29^. BepA is a member of the conserved M48 family of zinc metalloproteases and has two domains: a protease domain and a tetratricopeptide repeat (TPR) domain^27,30^. The protease domain contains a HEXXH motif responsible for zinc coordination, a conserved regulatory ‘plug’ with an additional histidine which coordinates zinc, and a dynamic, flexible ‘lid’^27,28,30,31^ (Supplementary Fig. 1b–e). The lid and plug occlude the active site of BepA (Supplementary Fig. 1c), and movement of both is thought to be needed for BepA to cleave its substrates^28^.

How BepA binds BAM, recognises its OMP substrates and catalyses OMP degradation are poorly understood. Here, we set out to determine how BepA binds BAM and performs its central role in OM proteostasis. Using cryoEM, we solve a series of BAM-BepA structures, both reconstituted in vitro and purified directly from the bacterial OM. The results reveal how the periplasmic BepA protease can seek out its clients at BAM for cleavage. Upon binding to BAM, the usually water-soluble helical lid of BepA buries into the membrane to reveal its active site, ready to cleave (mis)folding OMPs. We use AlphaFold3 (AF3)^32^ to search for sequence motifs required for BepA recognition, plug movement and cleavage, discovering and experimentally validating a sequence preference for Ar-X-Ar (where Ar = aromatic residue and X = any residue) motifs that are known to be enriched in OMP sequences^33^. Together, the results reveal the choreography required for OMP cleavage at the OM by BepA and suggest how BepA and BAM coordinate to resolve OMP-folding-induced stress at the OM.

## Results

### BepA binding induces a large conformational change in BAM

**Fig. 1:**
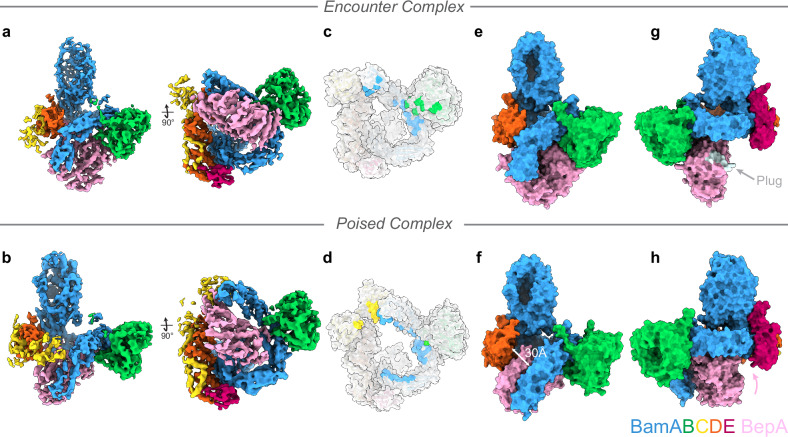
BAM-BepA complexes. CryoEM density map of (**a**) the Encounter Complex: BepA bound BAM with no conformational change and (**b**) the Poised Complex: BepA bound BAM in a different conformation. The BepA interaction interface within the periplasmic ring of BAM changes from (**c**). POTRA domains 1–3 and BamB in the Encounter Complex to (**d**). POTRA domains 1–5, BamB and BamC in the Poised Complex. (BepA is removed from (**c**, **d**) for clarity and regions directly interacting with BepA are shown in their protein colour. Supplementary Fig. 3. shows residues involved in hydrogen bonds and salt bridges in the interaction interface for **c**, **d**). **e** In the Encounter Complex: BepA bound BAM maintains the contacts between BamD and BamA POTRA-2, (**f**), but in the Poised complex BepA induces a change in conformation of BAM, breaking a salt bridge between POTRA-2 and BamD (residues R162 and D29, not shown) and resulting in a 30 Å separation of POTRA-2 from BamD. (BamC was removed for clarity in **e**, **f**). This allows BepA to move its active site upwards from below the periplasmic ring (**g**) to near the BamA barrel **h**. The Encounter Complex is shown in (**a**, **c**,** e** and **g**) while (**b**, **d**, **f** and **h**) display the Poised Complex.

A previous study^30^ using site-specific in vivo photo-crosslinking showed that BepA interacts with BamA, BamC and BamD, with subsequent Alphafold2 (AF2) models suggesting that BepA interacts with BAM within its periplasmic ring^34^. Therefore, we set out to determine the cryoEM structure of BAM-BepA. BAM and BepA were purified separately from the OM and periplasm of *E. coli*, respectively (Methods). BAM was then mixed with a 5-fold molar excess of BepA, and cryoEM was used to analyse any complexes that had formed. The results revealed two distinct conformations of BAM-BepA (Fig. 1a, b and Supplementary Fig. 2). In both structures, BepA is interacting within the periplasmic ring of BAM where BepA forms extensive interactions with BamA POTRA domains-1-3 and BamB (Fig. 1c, d and Supplementary Fig. 3). In one of the structures there is an increase in interactions between BAM and BepA such that BepA also interacts with BamA POTRA domains 4 and 5, and BamC (Fig. 1d and Supplementary Fig. 3d–f) increasing the interface area from 766Å^2^ to 1789Å^2^.

In the first conformation of BAM-BepA, BAM adopts the ‘Lateral Open’ state observed previously in many studies (Fig. 1e)^15,18,20,21,35–37^ (and is also the same conformation as apo-BAM present in this dataset (Supplementary Fig. 2e)), whilst BepA adopts the structure observed previously by crystallography^30,38^ and predicted by AF3 (Supplementary Fig. 1c). We therefore term this the ‘Encounter Complex’ as BepA appears to have bound BAM with no conformational rearrangements observed in either partner. In the second conformation, BepA binding has induced a different conformation in BAM where POTRA-2 and BamD no longer interact with each other, with BepA nestling between them (Fig. 1f), allowing the second helix grip domain of BamC to bind to BepA (Fig. 1b). In this state, a salt bridge that is observed in apo-BAM and in the Encounter Complex between residues R162 and D29 in POTRA-2 and BamD, respectively, has been broken, and there is a 30 Å movement of POTRA-2 away from BamD (Fig. 1f and Supplementary Movie 1). The movement enables BepA to increase its interactions within the periplasmic ring such that it now also interacts with POTRA domains 4 and 5 (Fig. 1b, d, f and Supplementary Fig. 3d–f). This moves the protease domain of BepA closer to the membrane, such that is now positioned within the periplasmic ring of BAM directly beneath the BamA barrel where OMP folding occurs (Fig. 1g, h and Supplementary Movie 1). Therefore, we term this the ‘Poised Complex’.

### The N-terminal region of BepA is required for its function

**Fig. 2:**
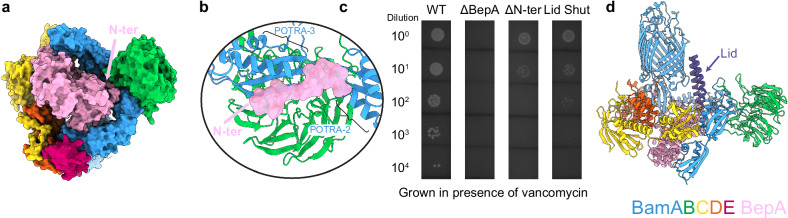
BepA’s unstructured N-terminal region and dynamic lid are functionally important. Interaction of BepA’s N-terminal residues with BamA POTRA-2, POTRA-3 and BamB shown (**a**) from a view looking from the periplasmic side up into the barrel of the Poised Complex and (**b**) from a view displayed from inside the periplasmic ring facing towards BamB (POTRA-2, POTRA-3, BamB and BepA’s N-terminal residues only shown for clarity). (Supplementary Fig. 3 shows details of interacting residues **c**). Representative growths of bacteria (Δ*bepA*) complemented with plasmids expressing WT BepA, untransformed control (ΔbepA), BepA(Δ*N-ter*) where residues 28–40 are deleted, Lid shut BepA(A64C, A173C). Complemented strains were grown in the presence of vancomycin (200 μg/ml) to probe OM permeability defects. (Supplementary Fig. 6 shows that disulphide bond of Lid shut BepA is fully formed and its location in the structure). **d** AF3 prediction of a BAM-BepA complex coloured by protein showing the BepA lid (purple) extending as a helix-turn-helix which interacts with the membrane-interacting loop of POTRA-3 and turn-6 of the BamA barrel.

In both the Encounter and Poised Complex, the N-terminal residues of BepA, which are unstructured in both AF3 predictions of the BepA structure alone (Supplementary. Fig. 1c) and in its crystal structure (PDB 6SAR^38^), interact with BamA POTRA-2, POTRA-3 and BamB (Fig. 2a, b and Supplementary Fig. 3). This interaction is also observed in AF3 models of BAM-BepA, suggesting co-evolution between these proteins. Interestingly, examination of BAM complexes from other World Health Organisation (WHO) priority pathogens shows that diderm bacteria, which lack BamB, also have no BepA homologue (Supplementary Fig. 4), supporting our finding that the N-terminal region of BepA is important for its interaction with BAM. To confirm the biological relevance of this interaction, we deleted the N-terminal residues from BepA (residues 28-40, residues 1–27 represent the signal sequence, which is removed by the signal peptidase during secretion into the periplasm) and a plasmid encoding BepA(ΔN-ter) was used to complement a Δ*bepA E. coli* strain. Both the Δ*bepA* strain and the deletion strain complemented with BepA(ΔN-ter) had compromised OMs, as evidenced by their inability to grow in the presence of vancomycin (Fig. 2c). Interestingly, the growth defect observed for the BepA(ΔN-ter)-complemented strain was not as severe as Δ*bepA E. coli*, suggesting some residual BepA function in the absence of the N-terminal region. This OM defect was rescued when the Δ*bepA* strain was complemented with a plasmid expressing full-length BepA^29,39^ (Fig. 2c). Combined, this suggests that the N-terminal region is important for BepA function.

### Shutting the lid inhibits BepA

Determining the structures of BAM-BepA required a large dataset ( ~ 89,000 movies), presumably because the two bound states are in dynamic equilibrium, which resulted in blurring of the cryoEM density of BepA. Extensive classification and focused classification were required to observe BepA in both the Poised and Encounter Complexes, so that the global resolution of the structures was worsened to > 5 Å but the b-factor remained unchanged (Supplementary. Fig. 2). However, in both complexes BepA could be rigid body docked into the maps, apart from its lid region which was poorly resolved (Supplementary Fig. 5a, b). AF3 models have the lowest confidence (low plDDT score) when predicting the lid region (Supplementary Fig. 5c, d) and the lid is either partially modelled (Supplementary Fig. 5e) or absent (Supplementary Fig. 5f) in its crystal structures^27,30^. In some AF2 predictions of BAM-BepA complexes (but not the top-ranked model) suggest that the lid region interacts with the BamA β-barrel, extending as a helix-turn-helix that would embed into the membrane alongside the BamA barrel^34^, a finding supported here by some AF3 models (but again, not the top-ranked predictions) (Fig. 2d and Supplementary Figs. 4, 5g, h). Indeed, DeepTMHMM^40^, a membrane helix prediction algorithm, also suggests that the lid sequence could comprise transmembrane helices (Supplementary Fig. 5j). Together, these findings suggest that the lid region of BepA is dynamic and could switch between conformations in which it is docked against the enzyme active site, is undocked in solution, and, when bound to BAM, adopts a membrane-embedded state.

To test the importance of the lid region dynamics in BepA function, we engineered a disulphide bond to lock the lid shut to the TPR domain (BepA(A64C, A173C)) (Supplementary Fig. 6). A plasmid encoding this Lid shut BepA mutant was used to complement the Δ*bepA* strain. Lid shut BepA also shows a compromised OM (Fig. 2c), confirming that lid dynamics are important for BepA function.

### Engaging the lid of BepA with BamA

**Fig. 3:**
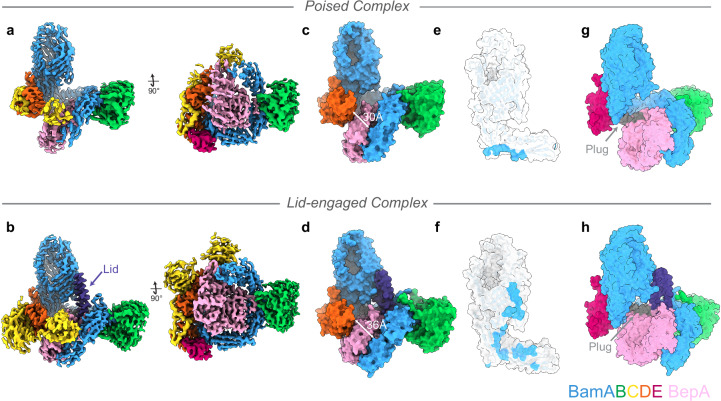
BepA’s lid engages with BAM. **a** CryoEM density map of the Poised Complex with an in vivo derived disulphide (D211C (BamA) and R155C (BepA)) between POTRA-3 of BamA and the lid of BepA (named here Poised^SS^). It represents the same structure as (Fig. 1b), and the Lid of BepA is unresolved. **b** CryoEM density map of the Lid-engaged Complex with an in vivo derived disulphide (P723C (BamA) and L186C (BepA)) between turn-6 of BamA and the lid of BepA. BepA’s lid forms two helices in the micelle. **c** In the disulphide bonded Poised complex, a 30 Å separation of POTRA-2 from BamD is observed, identical to that seen without the disulphide bond. **d** In the Lid-engaged Complex, an even larger separation of 36 Å is observed between POTRA-2 and BamD. Note that BamC is removed for clarity in (**c**, **d**). The BepA interaction interface with BamA changes from (**e**). POTRA-4 and the base of POTRA-5 in the Poised Complex to (**f**). closer to the membrane on POTRA-4, more extensively with POTRA-5 and with the BamA barrel in the Lid-engaged Complex. (Only the BamA barrel and POTRA-4,−5 are shown in (**e**, **f**), and regions directly interacting with BepA are highlighted in blue, Supplementary Fig. 8. shows residues involved in hydrogen bonds and salt bridges in the interaction interface for **e**, **f**). **g** The plug of the BepA active site is docked against the active site, inactivating the enzyme in both (**g**) the Poised Complex and (**h**) the Lid-Engaged complex. However, the plug (grey) moves closer to the membrane and the base of the BamA barrel in the Lid-Engaged complex. (BamD and BamC were removed for clarity in **g**, **h**). The Poised Complex is shown in (**a**, **c**, **e**, and **g**) while (**b**, **d**, **f**, and **h**). are the Lid-engaged Complex.

The AF3 prediction of BepA-BAM suggested that the lid could interact with the membrane-interacting loop of POTRA-3 (Fig. 2d and Supplementary Fig. 5k). Such a movement from solvent exposed to membrane embedded could be important in activating BepA for OMP cleavage. To test this hypothesis, we introduced Cys residues into the lid of BepA (R155C) and the relevant loop of POTRA-3 (D211C) and purified the BAM-BepA complex directly from *E. coli* membranes using a Strep-tag on BepA (Methods). Analysis by SDS PAGE showed that a disulphide bond between BepA and BamA had formed in vivo (Supplementary Fig. 7a). A smaller cryoEM dataset (11,044 movies) of this sample contained a single BAM-BepA conformation (Fig. 3a and Supplementary. Fig. 7) and the resulting structure revealed the same Poised Complex as that observed in the sample formed in vitro which lacks Cys residues (i.e., POTRA-2 moves 30 Å away from BamD and the second helix grip domain of BamC binds to BepA (Fig. 1b)). The increase in occupancy of BepA resulted in a dramatic increase in the resolution of the structure to 3.9 Å which allowed an atomic model of BepA to be built. However, once again, the lid region appeared to be dynamic and could not be resolved in the structure, suggesting that even when captured by disulphide bonding to the loop of BamA POTRA-3, the lid remains dynamically disordered.

Given the lack of a resolved lid in this structure, a new cysteine pair was engineered, but this time the disulphide link was placed between the BepA lid (L186C) and turn-6 of the BamA β-barrel (P723C) which are also predicted to be in close contact in the AF3 model (Fig. 2d and Supplementary Fig. 5k). Again, the proteins were co-expressed and purified as a disulphide bonded complex directly from *E. coli* membranes using BepA’s Strep-tag (Supplementary Fig. 8a) and analysed using cryoEM (Supplementary Fig. 8). The resulting structure (Fig. 3b) revealed the lid fully resolved in the micelle as a pair of helices alongside an ordered lipid or detergent molecule (Supplementary Fig 8j). This confirms the AF3 model and transmembrane helix predictions that the lid can indeed insert into a membrane in an unusual (for the OM) helical conformation (Fig. 2d and Supplementary Fig. 5g–k). This dataset similarly has a single conformation of BAM-BepA, which we term the ‘Lid-engaged’ complex. In this conformation, BepA has induced an even greater separation between BamA and BamD from 30 Å in the Poised complex (Fig. 3c) to 36 Å (Fig. 3d and Supplementary Movie 2). The second helix grip domain of BamC interacts with the TPR domain of BepA in the same location between the two structures (Fig. 3a, b and Supplementary Movie 2). The increase in diameter of the periplasmic ring allows BepA to interact even more extensively with POTRA-4 and POTRA-5, with the lid moving up into the membrane, where it also interacts with the BamA barrel, increasing its interaction area with BamA from 1789Å^2^ to 2748Å^2^ (Fig. 3e, f and Supplementary Fig. 9). Importantly, these coordinated movements place the BepA active site closer to the membrane (Fig. 3g, h).

**Fig. 4:**
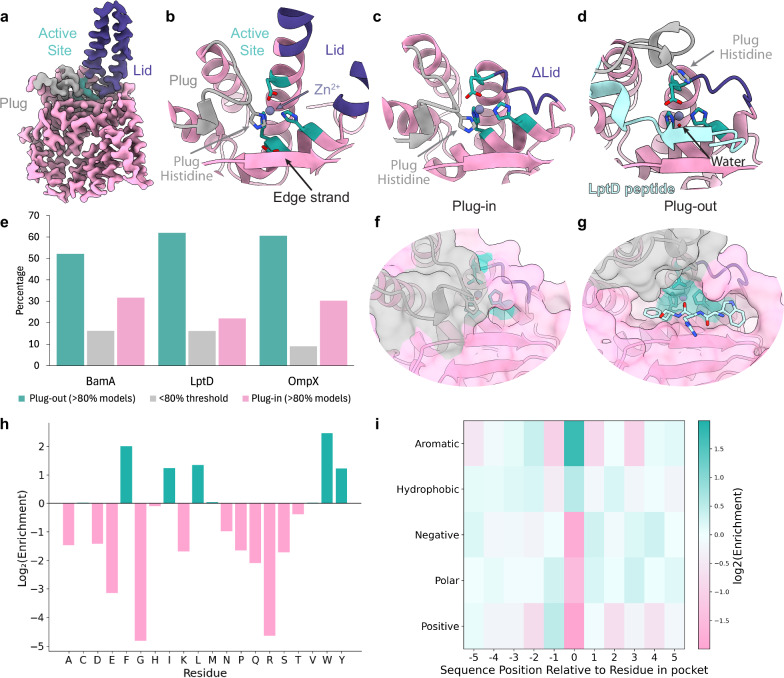
BepA binds OMP peptide sequences enriched in Ar-X-Ar motifs. **a** CryoEM density for BepA in the Lid-engaged Complex where the lid of BepA has formed two helices that project into the membrane. **b** BepA lid-engagement with the membrane/BamA exposes the edge strand of BepA, but the plug histidine (His246) still coordinates the zinc ion. **c** AF3 prediction of BepA(Δlid) (residues 150-199 were deleted for the prediction) with a plug-in conformation in which the plug histidine (residue H246 in the native sequence) coordinates the zinc. **d** AF3 prediction of BepA(Δlid) in the presence of LptD peptide (321-MDQVWRFNVDYTKVSDPSYF-340) (cyan) in a plug-out conformation in which the plug histidine no longer coordinates the zinc and is replaced by a water molecule. **e** Percentage of AF3 BepA predictions in a Plug-in or Plug-out conformation modelled with 350 15-residue peptides from BamA, LptD and OmpX. Green is plug-out peptides, pink is plug-in peptides and grey is peptides that did not meet the 80% threshold. **f** Surface representation of AF3 model of BepA(Δlid) in a plug-in conformation. **g** Surface representation of AF3 models of BepA(Δlid) with Trp-Arg-Phe from LptD peptide bound to the plug-out conformation. Plug undocking reveals a pocket adjacent to the zinc ion containing the bound Phe and the previous carbonyl that would be attacked during catalysis. **h** Residues enriched in the revealed pocket in plug-out BepA across all AF3 predictions. (green is residues enriched, pink is residues not enriched). **i** Heatmap to showing the class of residues enriched in this pocket and 5 residues N/C-terminal to this site in the bound sequences.

The engagement of the BepA lid with the membrane and the BamA barrel exposes the active site of BepA, revealing its so-called ‘edge strand’ (Fig. 4a, b), which has been shown previously to interact with folding LptD^28^. This suggests that the Lid-engaged complex represents a state of BepA that is primed for OMP cleavage. However, this conformation of BepA would be inactive as all Zn^2+^ coordination sites are occupied with protein ligands: it is coordinated by three His and a Glu (H136, H140, E201 and H246) and there would be no site for a catalytic water molecule required for proteolysis (Fig. 4b). Hence, additional dynamic rearrangements are required before catalysis of peptide bond cleavage can occur.

### Moving the plug of BepA

The structure of the Lid-engaged complex shows that interaction of BepA with BAM alone does not trigger plug movement, at least in the conditions sampled herein, raising the question of how BepA is finally activated for OMP cleavage, as this would require the plug to disengage the His246 from Zn^2+^ at the active site (Fig. 4b and Supplementary Fig. 1e) to allow a catalytic water molecule to bind. One possibility is that substrate binding to BepA might trigger this movement. To test this hypothesis, we used AF3 to explore which sequences within OMPs might enable plug movement when interacting with BepA. To do this, we removed the lid (residues 150–199) from the BepA sequence as the lid occludes the active site in all AF3 predictions of apo-BepA. AF3 models of apo-BepA(Δlid) resulted in a plug-in conformation of BepA for each of the five models generated, with the plug histidine (His246 in the native sequence) coordinated as one of the Zn^2+^ ligands (Fig. 4c). We next generated AF3 models of BepA with a peptide (MDQVWRFNVD**Y**TKVSDPSYF) from strand 7 of LptD that has previously been demonstrated to interact with the edge strand of BepA (using photoactivated crosslinking to Tyr-331 (shown in bold in the sequence above)^28^). Each of the five AF3 models of BepA(Δlid) with this peptide showed that the plug had moved such that the zinc ion was no longer coordinated to the plug histidine but was instead replaced by a water molecule that would be required for catalysis, and the LptD peptide forms a β-strand that hydrogen bonds to the BepA edge strand (Fig. 4d). This suggests that the plug can move in response to substrate interaction.

To probe this possibility further, we next used AF3 to predict the structures of BepA(Δlid) with 15-residue peptides encompassing the sequences of three OMPs from the *E. coli* OM: LptD, BamA and OmpX. A total of 350 peptides was created by moving a 15-residue sliding window along each sequence in 5- residue steps. The resulting predictions were analysed for plug movement (“Methods”). The results showed that ~ 60% of peptides generated at least 4 out of 5 models with the plug-out, while ~ 30% of peptides generated at least 4 predictions out of 5 where the plug did not move (Fig. 4e). This suggested a sequence specificity to binding/plug movement. In peptides that displaced the plug, 96% were found to interact with the exposed edge strand based on Cα contact analysis (Ca-Ca <5 Å (“Methods”)). DSSP analysis indicated that 25% of peptides adopted β-strand conformations, of which 85% were oriented antiparallel to the edge strand.

Adjacent to the zinc ion is a pocket that is exposed by movement of the plug (Fig. 4f, g). This pocket is occupied by Phe (residue 7) in our initial LptD peptide prediction and positions the preceding peptide substrate carbonyl group close to zinc ion and adjacent to the water molecule, thus indicating where BepA would likely cleave (Fig. 4g). We therefore determined the peptide residue that occupies this pocket in all our AF3 ‘plug-out’ models and this showed an enrichment of aromatic, Leu or Ile residues at that position (Fig. 4h). Analysing the surrounding sequence of this bound residue further revealed a strong preference for the motif Aromatic-Positive-Aromatic, and a slightly weaker preference for sequences where the aromatic residues could be hydrophobic residues (Fig. 4i). OMPs are enriched in Aromatic-X-Aromatic motifs^33^ suggesting BepA has a preference for this common OMP motif, potentially rationalising its ability to degrade stalled OMPs.

**Fig. 5:**
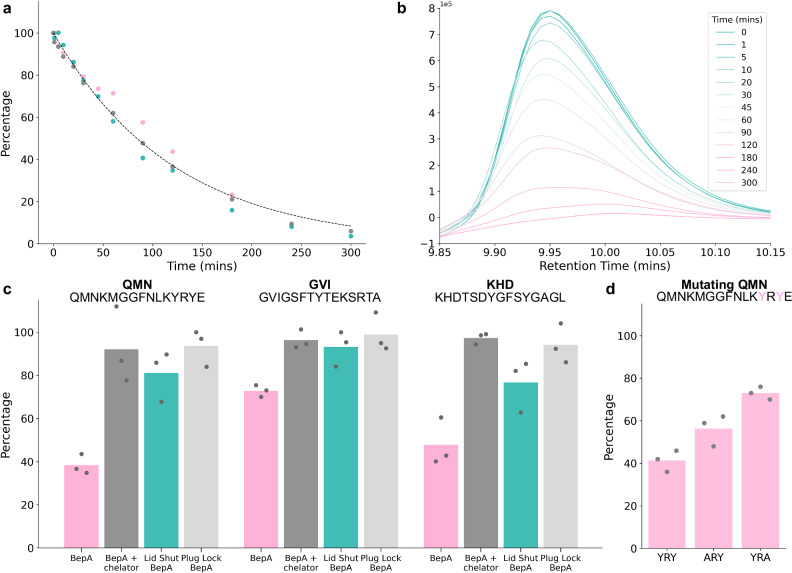
BepA degrades OMP peptides. **a** BepA mediated degradation of QMN peptide (QMNKMGGFNLKYRYE) over time. Fitted to an exponential. Replicates in different colours. **b** Raw HPLC traces of the peak of QMN peptide at different timepoints after incubation with BepA from 0 to 300 mins. **c** Percentage of QMN peptide, GVI peptide (GVIGSFTYTEKSRTA) or KHD peptide (YKHDTSDYGFSYGAG) remaining after two hours incubation with WT-BepA, WT-BepA in the presence of the zinc chelator, 1, 10 phenanthroline, Lid shut BepA or Plug-lock BepA. Triplicate data are shown. **d** Percentage of peptide remaining after two hours incubation with WT-BepA with different amino acid substitutions in the Ar-X-Ar motif in the QMN peptide where the sequence is either the WT sequence, designated YRY (QMNKMGGFNLKYRYE), or the variants ARY (QMNKMGGFNLKARYE) or YRA (QMNKMGGFNLKYRAE) in this motif. Triplicate data are shown.

To test BepA’s ability to cleave OMP peptides, we mixed BepA with a previously characterised peptide (with the sequence QMNKMGGFNLKYRYE; henceforth termed QMN) that forms β-strand 1 of folded OmpX^7^ and monitored peptide cleavage using HPLC detected using Tyr fluorescence (Methods). AF3 predictions of BepA(Δlid) with this peptide resulted in 80% of models in a plug-out conformation. The experiments revealed that BepA is able to cleave the QMN peptide (Fig. 5 and Supplementary Fig. 10), and degradation was prevented by the addition of the zinc-chelating agent 1, 10 phenanthroline (Methods). The addition of QMN peptide to Lid shut BepA or Plug lock BepA mutants (a disulphide (E103C, E241C) engineered to prevent plug movement based on previous studies^29,36^) did not result in peptide degradation (Fig. 5c). This shows that BepA degrades OMP peptides via a zinc-dependent mechanism that requires both lid and plug movement. Two additional OmpX-derived peptides (corresponding to β-strands 2 and 4 of natively folded OmpX, termed GVI and KHD, also characterised previously^7^) were also degraded by BepA, with zinc chelation, shutting the lid and stopping plug movement all preventing this degradation (Fig. 5c). Altering the Ar-X-Ar motif in the QMN peptide by changing each Tyr separately to Ala resulted in decreased degradation by BepA (Fig. 5d), indicating the importance of the Ar-X-Ar motif for BepA action.

## Discussion

In this study, we demonstrate how BepA is activated to degrade stalled OMPs on BAM, from its initial binding and induction of conformational changes in BAM, its lid-engagement with the membrane, and a substrate-binding-induced plug movement for proteolysis of OMP peptides. This builds on previous studies, which revealed that BepA can bind to BAM^27,28,30,31,34,38,39^, but left unresolved any structural mechanism of how BepA might bind and degrade stalled OMPs, or how BAM responds to BepA binding. Previous studies have also lacked details about the movements of the lid and plug of BepA required for proteolysis^31,38^ or the sequence specificity of BepA-mediated degradation.

The cryoEM structures here reveal an extensive interaction interface between BepA and BAM in which BepA forms contacts with all five POTRA domains of BamA, as well as with BamB and BamC, cementing the roles of these domains/proteins as docks for periplasmic partners. The interaction of BepA with BAM is reminiscent of the BAM-SurA interaction^11,12^ (Supplementary Fig. 11). In both cases, BepA/SurA initially binds BamA and BamB, and this then triggers the breakage of the salt bridge between R162 in POTRA-2 and D29 in BamD (which is observed in all apo-BAM structures) for the resulting conformational change in BAM. In the Encounter Complex, BepA interacts with three of the five POTRA domains (POTRAs-1 to −3), as well as with BamB within the periplasmic ring. However, the binding site for BepA is on the opposing side on POTRA-1 to the SurA binding site. This suggests that BepA could bind to BAM when SurA is also bound, but it is currently unknown whether such an event would be likely in vivo. The large conformational change in BAM that is induced by BepA binding in the Poised and Lid-engaged Complexes would move SurA’s OMP binding site further away from BamA-β1, potentially preventing or impeding delivery of an OMP to BAM. Equally, BepA’s interaction within the BAM periplasmic ring means it could act as a ‘stopper’, physically occluding delivery of an OMP to BamA-β1 for folding and membrane insertion. This could explain why the Lid-shut mutant of BepA does not display as severe a phenotype as BepA deletion, because locking the BepA lid shut would not impinge on this putative blocking role.

The lid of BepA that interacts with the membrane as a pair of helices gives BepA an extraordinary level of spatial control. Once BepA has bound to both BAM and the membrane, it is primed to reveal its active site, ready for dissociation of the inhibitory plug and activation of the enzyme, ready to cleave OMPs stalled in the act of BAM-catalysed folding. Several structures of OMPs stalled on BAM have been elucidated^11,13,14,18^. In each of these, BepA’s active site would sit below the nascent OMP barrels, ideally placed to cleave any unfolded OMP chain just below the membrane (Supplementary Fig. 12) (all BAM-OMP stalled structures contain regions of unresolved polypeptide chain in the constructs used^16^). The TPR domain of BepA is also positioned directly below the BamA barrel and, as previously proposed^30,38^, could contribute to OMP recognition by BepA, given the relatively small binding interface formed by the edge strand and active-site pocket. Whether/how BepA’s enzymatic activity is modulated by BAM remains unclear. When added to BAM in detergent micelles, BepA cleaved the QMN peptide similarly to BepA in the absence of BAM (Supplementary Fig. 10). Whether this is the case for other peptide sequences, for BAM-BepA in a native membrane environment, or for OMPs at different stages of folding remains unknown and warrants further investigation.

The fact that BepA can cleave OMP peptides in vitro without requiring BAM or membrane binding suggests that BepA could also degrade OMPs at earlier stages of the OMP biogenesis pathway. It also explains why deleting the N-terminal 12 residues of BepA, and thus the proper localisation of BepA to the BAM complex, does not display as severe a phenotype as BepA deletion, because BepA with reduced BAM binding may still be capable of degrading OMPs in the periplasm.

The observation that BepA recognises the same Ar-X-Ar motifs in OMPs that also drive SurA binding to unfolded OMPs;^7,33,41^ β-signal binding to BamA-β1^42–44^ and the activation of the sigmaE stress response (triggered when C-terminal Tyr-X-Phe motifs in unfolded OMPs accumulate in the periplasm and bind to DegS^45^) is striking. It suggests a mechanism by which BepA is able to cleave OMP sequences that have failed to fold efficiently. The prevalence of Ar-X-Ar motifs in these diverse processes suggests an emerging general role for this motif in periplasmic and OM homoeostasis.

Although membrane localisation is a relatively common spatiotemporal control event in biology^46^, the details of BepA activation are unusual for an enzyme activation mechanism. However, the ability to undergo a structural transition that inserts two usually solvent-exposed helices deep into a membrane is an unusual feature for an enzyme activation mechanism, especially in the OM, wherein transmembrane helices are rare. Membrane recruitment of enzymes with amphipathic helices, such as phospholipase A2 and phosphocholine cytidylytransferase, has been demonstrated^46^, but these interact with lipid head groups rather than inserting into or traversing the bilayer. The phenomenon is more commonly observed for proteins involved in the disruption of membranes, such as toxins^47^ or during apoptosis (e.g., Bax^48^).

In all the cryoEM structures reported here, BepA’s plug remains in its inhibitory ‘plug-in’ conformation. The data presented herein suggest that plug movement is induced by substrate binding rather than BAM interaction. The regulatory plug of BepA is a common feature of M48C proteases, including in *E. coli* HtpX, YcaL, LoiP and mitochondrial Oma1 (Supplementary Fig. 13), but evidence as to how their plugs move is currently lacking. It is tempting to suggest that substrate interaction could trigger plug movement in all these proteases, given the structural similarities of their protease domains.

In summary, we have revealed a mechanism for how BepA binds to BAM, becomes activated and leads to OMP cleavage, but many questions remain. These include how BepA distinguishes between BAM complexes with stalled OMPs and those that are productively folding OMPs into the OM. How BepA cleavage of OMPs at BAM resolves stalled complexes, especially OMPs that already have significant portions of their sequence integrated into the membrane as β-strands, also remains unclear. One possibility is that once cleaved, these OMPs are removed by Skp, which has been shown to be able to extract OMPs from BAM that contain up to six or seven strands that have already been folded on BAM^49^. It could also be that the engagement of BepA’s lid in the membrane enables it to interact with membrane-integrated OMPs to facilitate their removal from the membrane. Further work is needed to resolve these questions. Finally, how and whether BepA coordinates with other M48 proteases linked to OMP degradation, such as the lipoproteins YcaL and LoiP^29,50^ remains an open question. The structures of BepA with BAM reported here demonstrate a distinct protease activation mechanism that is likely common amongst related pathogenic diderm bacteria (Supplementary Fig. 4). Antimicrobial design might now be possible, via inhibition of BepA binding to BAM or to its substrates, or prevention of either lid or plug movement, targeting this essential protease-mediated rescue of OMP folding.

## Methods

### Strains and plasmid construction

Details of plasmids, primers and strains used in this study are given in Supplementary Tables 1, 2 and 3. BepA plus its RBS was amplified out of *E. coli* BW25113 with flanking cut sites and cloned into pSCRhaB2. A GSS followed by a TwinStrep tag was inserted to the C-terminus of pSCRhaB2-BepA using Q5 site-directed mutagenesis (NEB) to generate pSCRhaB2-BepA_CTTS_. All site-directed mutagenesis was performed using Q5 site-directed mutagenesis (NEB). Gibson Assembly was used to generate pZA31-BepA.

### Protein expression and purification

#### BepA and BepA mutants

pSchraB2-BepA_CTTS_ or mutant plasmids containing relevant mutations (Supplementary Table 1) were transformed into BL21(DE3) and selected with 10 μg/ml trimethoprim (Formedium). Single colonies were used to inoculate 10 ml LB in 25 ml glass culture tubes, which were cultured at 37 °C, 220 rpm overnight. These overnights were then used to inoculate 2x 1 L LB in 2 L baffled flasks supplemented with 10 μg/ml trimethoprim to a starting OD_600_ of 0.05. and the bacteria cultured at 37 °C to an OD_600_ of 0.6 before inducing protein expression with 0.4% rhamnose for 1 hour. Cells were harvested by centrifugation at 7000 x *g*, 10 min, 4 °C in a JLA8.1000 rotor, and the pellet stored at − 20 °C. Pellets were resuspended in 50 ml 20 mM Tris-HCl, pH 8 (Fisher) with EDTA-free protease inhibitor tablets (Merck), homogenised at 8000 rpm, then lysed twice at 30kPSI in a cell disruptor (Constant Systems). The lysate was centrifuged at 20,000 x *g*, 4 °C, 30 min in a JA25.50 rotor. The supernatant was filtered using 0.45 µm membrane (Merck), then loaded onto a 1 ml StrepTrap XT Column (Cytiva) equilibrated with Strep Wash Buffer (20 mM Tris-HCl pH8, 150 mM NaCl). The column was washed with 10 column volumes of Strep Wash Buffer, then eluted into 5 ml fractions using Strep Wash Buffer supplemented with 50 mM biotin (Merck). The eluted protein was pooled, concentrated using a 30 kDa MWCO Vivaspin20 centrifugal concentrator (Sartorius) and further purified using a Superdex 75 column on an ÄKTA protein purification system in 20 mM Tris-HCl, pH 8.0, 150 mM NaCl. BepA-containing fractions were pooled and concentrated as above to 50 µM–150 μΜ before being snap frozen in liquid nitrogen and stored at − 80 °C.

#### BAM complex (described previously^11^)

pTrc99a-BamABCDE_CT8His_ was transformed into BL21(DE3) cells and selected with 100 µg/ml ampicillin (Melford). Single colonies were used to inoculate 100 ml LB in 250 ml baffled conical flasks, which were cultured at 37 °C, 220 rpm overnight. These cultures were pooled and then inoculated into 10x 1 L LB in 2 L baffled flasks supplemented with 100 µg/ml ampicillin to a starting OD_600_ of 0.05. Cultures were grown to an OD_600_ of 0.6–0.8 before protein expression was induced with 1 mM IPTG, and cultures were allowed to grow for a further 60–90 min. Cells were harvested by centrifugation at 7000 x *g*, 10 min, 4 °C in a JLA8.1000 rotor, and the pellet stored at − 20 °C. Pellets were resuspended in 100 ml 20 mM Tris-HCl pH 8 with EDTA-free protease inhibitor tablets, homogenised at 8000 rpm, then lysed twice at 30kPSI in a cell disruptor. The lysate was centrifuged at 20,000 x *g* at 4 °C for 15 min in a JA25.50 rotor, and the supernatant then centrifuged at 210,000 x *g* at 4 °C for 30 min in an MLA-50 rotor. Membrane pellets were resuspended in 60 ml 20 mM Tris-HCl pH8, 150 mM NaCl, 1% (w/v) DDM and broken up with 30 strokes in a Dounce homogeniser before incubating for 30 minutes with agitation at 4 °C. The sample was centrifuged again at 210,000 x *g*, 4 °C, 30 min in an MLA-50 rotor, the supernatant was retained and doped with imidazole (Acros Organics) to a final concentration of 20 mM. The sample was loaded onto a 5 ml HisTrap FF column (Cytiva) equilibrated in Wash buffer (20 mM Tris-HCl, pH 8, 150 mM NaCl, 0.05% (w/v) DDM) supplemented with 20 mM imidazole. The column was washed with 5 column volumes of Wash buffer with 20 mM imidazole before the bound protein was eluted with 4 Column Volumes of Wash buffer supplemented with 500 mM imidazole. The eluted protein was pooled, concentrated to ~ 5 ml in a 100 kDa MWCO Vivaspin20 centrifugal concentrator (Sartorius). Finally, the protein was further purified by size exclusion using a Superdex 200 16/600 pg gel filtration column on an ÄKTA protein purification system. BAM-containing fractions were pooled and concentrated as above to 50 µM before being snap frozen in liquid nitrogen and stored at − 80 °C.

#### Disulphide bonded BepA-BAM complexes

pTrc99a-BamABCDE_CT8His_ and pSChraB2-BepA_CTTwinStrep_ containing relevant cysteine variants (Supplementary Table 1) were co-transformed into BL21(DE3) and selected with 10 μg/ml trimethoprim and 100 µg/ml ampicillin. Single colonies were used to inoculate 100 ml LB in 250 ml baffled conical flasks, which were cultured at 37 °C, 220 rpm overnight. These cultures were pooled and then inoculated into 10x 1 L LB in 2 L baffled flasks supplemented with 100 µg/ml ampicillin and 10 μg/ml trimethoprim to a starting OD_600_ of 0.05. Cultures were grown to an OD_600_ of 0.6 before protein expression was induced with 1 mM IPTG and 0.4% rhamnose, and cultures were allowed to grow for a further 60 min. Cells were harvested by centrifugation at 7000 x *g*, 10 min, 4 °C in a JLA8.1000 rotor. Cells were pooled and resuspended in 1 L of PBS (OXOID) before 0.2 mM 4-DPS (Sigma Aldrich) was added from a 20 mM stock in DMSO and incubated with stirring for 1 h. Cells were pelleted again by centrifugation at 7000 x *g*, 4 °C, 10 min and stored − 20 °C. Pellets were resuspended in 100 ml 20 mM Tris-HCl pH 8 with EDTA-free protease inhibitor tablets, homogenised at 8000 rpm, then lysed twice at 30kPSI in a cell disruptor. The lysate was centrifuged at 20,000 x *g* at 4 °C for 15 min in a JA25.50 rotor, and the supernatant then centrifuged at 210,000 x *g* at 4 °C for 30 min in an MLA-50 rotor. Membrane pellets were resuspended in 30 ml 20 mM Tris-HCl pH8, 150 mM NaCl, 1% (w/v) DDM and broken up with 30 strokes in a Dounce homogeniser before incubating for 30 min with agitation at 4 °C. The sample was centrifuged again at 210,000 x *g* at 4 °C for 30 min in an MLA-50 rotor, and the supernatant loaded onto a 1 ml StrepTrap XT Column (Cytiva) equilibrated with Strep Wash Buffer (20 mM Tris-HCl pH8, 150 mM NaCl). The column was washed with 10 column volumes of Strep Wash Buffer, then eluted into 1 ml fractions using Strep Wash Buffer supplemented with 50 mM biotin (Merck). The eluted protein was pooled, concentrated to ~ 5 ml in a 100 kDa MWCO Vivaspin20 centrifugal concentrator (Sartorius). Finally, the protein was further purified by size exclusion using a Superdex 200 gel filtration column on an ÄKTA protein purification system. Complex-containing fractions were pooled and concentrated to ~ 2–4 mg/ml using Vivaspin 20 100 kDa MWCO centrifugal concentrators (Sartorius) and applied directly onto grids for cryo-EM analysis. (For the barrel-crosslink purification, 50 mM EDTA pH8 was added to all buffers.)

### CryoEM

#### Grid preparation

BAM and BepA were mixed at a molar concentration of 1:5 before being applied to the grid to a final concentration of 2 5μΜ ΒΑΜ and 125 μΜ BepA. BAMxBepA crosslinked samples were applied directly to the grid at ~ 2–4 mg/ml. 4 μl samples were applied to R1.2/1.3 (300 mesh) Quantifoil grids, previously plasma cleaned for 60 s using a Tergio Plasma Cleaner (PIE Scientific). Grids were blotted for 5-6 sec at 4 °C and > 90% humidity and plunge-frozen in liquid ethane with a Vitrobot Mark IV 480 (ThermoFisher).

#### Data collection and processing

Datasets were collected on a 300 keV Titan Krios electron microscope (ThermoFisher) in the Astbury Biostructure Laboratory operated with a Selectris energy filter operating with a 10 eV slit and a Falcon4i detector in counting mode. All datasets were collected with nominal defocus range of − 0.9 to − 3.0 µm. Data acquisition parameters for each dataset can be found in Supplementary Table 5. All processing was performed using Relion4 or 5^51^, unless otherwise stated. Movies were motion corrected and dose-weighted. CTF parameters were estimated with CtfFind4.1^52^.

#### BAM-BepA dataset

Particles were initially picked with crYOLO (v1.8)^53^ using the general model^53^, which yielded 6.6 million particles. One round of 2D classification using the VDAM algorithm was used, and only 2D classes with BAM-like features were selected to remove the BepA only particles yielding 2.6 million particles. A second round of 2D classification using the EM algorithm was performed, and 1.6 million particles were taken forward to 3D. An initial model was generated, and particles were subjected to a 3-class 3D classification. One class of 750,000 particles was then furthered classified into 7 classes. 3 of these classes were refined to resolutions where secondary structure could be observed. The first corresponded to apo-BAM, and this was iteratively refined to a resolution of 3.9 Å. The other two corresponded to BAM-bound BepA structures. These classes of 92,000 and 89,000 particles were initially refined to 4.8 Å and 4.4 Å but the density for BepA in both was poor with b-factors of − 236 and − 204, respectively. All refinements were performed with Blush^54^. In the Encounter Complex class, a 3-class focused classification with a T value of 8 was performed with a mask around POTRA1-4, BamB and BepA, the class with the most BepA density containing 8000 particles was refined to a global resolution of 5.3 Å and a b-factor of − 170. In the Poised Complex class, a 3-class focused classification with T of 8 was also performed, the largest and best class of 72,000 particles was refined again to the same resolution of 4.8 Å with a b-factor of − 231. A second focused classification, again with 3 classes and *T* = 8, was performed with a mask around BepA and POTRA-5. The largest class of 47,000 particles was refined again to a resolution of 5.3 Å and a b factor of − 226. The global resolution of both these structures worsened, but the b-factor either was unchanged or slightly improved, and there was an improvement of the BepA density such that BepA could be rigid body fit into the densities. (Processing Diagram in Supplementary Fig. 2)

#### BAM-BepA dataset (POTRA-3 crosslink) – Poised Complex

Particles were initially picked with crYOLO (v1.8) using the general model^53^, which yielded 1.3 million particles. One round of 2D classification using the VDAM algorithm was used, yielding 619,000 particles that were taken forward for 3D classification. An initial model was generated, and particles were subjected to a 5-class 3D classification. The largest class of 249,140 particles was iteratively refined with Blush with rounds of CTF refinement and Polish and a final round of Non-Uniform refinement in Cryosparc^55^ to a resolution of 3.9 Å. (Processing Diagram in Supplementary Fig. 7)

#### BAM-BepA dataset (barrel crosslink) – Lid-engaged Complex

Particles were initially picked with crYOLO (v1.8) using the general model^53^, which yielded 4.2 million particles. One round of 2D classification using the VDAM algorithm and a second round using the EM algorithm yielding 666,936 particles that were taken forward for 3D classification. An initial model was generated, and particles were subjected to a 5-class 3D classification. The largest class of 226,760 particles was subjected to a 7-class 3D classification, and the four highest resolution classes were iteratively refined with Blush with rounds of CTF refinement and Polish and a final round of Non-Uniform refinement in Cryosparc^55^ to a resolution of 3.6 Å. (Processing Diagram in Supplementary Fig. 8)

#### Model building

The appropriate AF3 BepA model (either with lid-up or down) was rigid-body docked into all maps in ChimeraX^56^. 5LJO minus POTRA-1 to − 3 and BamB, and a separate model of POTRA-1 to −3  and BamB from 5LJO were rigid-body fit into the maps. The models encompassing all proteins were then saved as a single model. The model was optimised in ISOLDE^57^ and then iteratively refined using real space refinement in PHENIX1.21^58^ and manual refinement in COOT^59^ until satisfactory geometry and fit between model and map assessed using MolProbity^60^ Details of initial models used for each structure and model building statistics for all structures are in Supplementary Table 6. Models for the BAM-BepA dataset were only rigid body fit due to the low resolution of maps.

### In vivo complementation assay

BWT255 ΔBepA (Keio collection, Horizon Discovery) was transformed with plasmids pZA31-BepA or a mutant variant (Supplementary Table 1). Colonies were inoculated into 10 ml LB with 50 µg/ml kanamycin and 25 µg/ml chloramphenicol (omitted for untransformed controls) and grown overnight at 37 °C in 25 ml glass culture tubes shaking at 220 rpm. The following day the OD_600_ of each culture was measured, and 10 ml fresh LB + supplements in 25 ml glass culture tubes was inoculated to a starting OD_600_ of 0.05. Cultures were then grown at 37 °C, 220 rpm to an OD_600_ of ~ 0.6. Culture ODs were then normalised to OD_600_  =  0.1, and serial dilutions made into LB only. 2 µL of each of these dilutions was then spotted onto LB  +  1.5% (w/v) agar plates containing 200 µg/ml vancomycin (Formedium). These were allowed to dry, and the plate then incubated overnight at 37 °C. Plates were imaged in a Uvitec Alliance Q9 imaging system.

### AF3 BepA(Δlid) with 15mer peptides

AF3^32^ installed on a local workstation was used to predict BepA(Δ1-40, 150–199) (the lid and N-terminus were removed for all predictions) in the presence of Zn^2+^, H_2_O and 15-residue peptides. The 15-residue peptides encompassed the whole sequence of LptD, BamA and OmpX, moving along each sequence by 5 residues, totalling 350 peptides. 5 models were predicted per peptide. For each model, the distances from the zinc ion to both NE2 and ND1 of the regulatory histidine were calculated using Biopython^61^. Models where both distances were greater than 3 Å were designated plug-out conformation

In plug-out models, peptide interactions with the exposed edge strand were identified using a Cα–Cα distance-based contact analysis, where peptides containing residues within 5 Å of residues 64–69 (corresponding to residues 105–110 in the BepA sequence) were classified as interacting. β-strand formation was assessed using DSSP secondary structure assignment (mkdssp^62^, Sali lab implementation), accessed via Biopython. Strand orientation was determined from the relative direction of backbone Cα vectors, allowing classification as parallel or antiparallel.

For each plug-out model, the residue closest to the valine in the pocket were identified using Biopython (Val-92 in AF3 predictions corresponding to Val-133 in BepA sequence). The composition of residues in this pocket was analysed to determine enrichment relative to the background sequence. For each amino acid, enrichment was calculated as the log_2_ ratio of the observed frequency to the background frequency. Residues were then grouped into chemical class, and a sequence window +/− 5 residues around the identified residue was extracted. Enrichment was calculated as a log_2_ ratio of observed to background frequencies and visualised as a heatmap.

### OMP peptide proteolysis

Peptides were solubilised in 100% DMSO at > 1 mM and diluted to 100μΜ in 20 mM Tris-HCl pH8, 150 mM NaCl. 10 μM peptide was added to 3μΜ ΒepA (or mutant). The reaction was stopped at a given time point (0, 2, 5, 10, 20, 30, 45, 60, 90, 120, 180, 240 or 300 min) using 10 mM 1,10 phenanthroline (Merck). The samples were analysed by Reverse-phase HPLC on a Shimadzu Nexera LC-40 system. 10 μl of sample was injected onto a C8 column (Phenomenex) using a linear gradient from 5-95% acetonitrile in 0.1% TFA over 20 min with detection of tyrosine fluorescence (Ex 275 nm, Em 305 nm). QMN peptide = QMNKMGGFNLKYRYE^7^, GVI peptide = GVIGSFTYTEKSRTA^7^, KHD peptide = KHDTSDYGFSYGAGL^7^, ARY = QMNKMGGFNLKARYE^7^, YRA = QMNKMGGFNLKYRAE^7^. For samples comparing the QMN peptide mutation (QMN, ARY, YRA), samples were analysed in the absence of phenanthroline.

### Reporting summary

Further information on research design is available in the Nature Portfolio Reporting Summary linked to this article.

## Supplementary information

Supplementary Information

Description of Additional Supplementary Files

Supplementary Movie 1

Supplementary Movie 2

Reporting Summary

Transparent Peer Review file

## Source data

Source Data

## Data Availability

CryoEM reconstructions and corresponding coordinates have been deposited in the Electron Microscopy Data Bank (EMBD) and the Protein Data Bank (PDB), respectively: Encounter Complex EMD-5655028JR, Poised Complex 5655928JU, Disulphide bonded Poised Complex EMD-5654928JQ, and Lid-engaged Complex EMD-5654328JL. The raw cryoEM datasets used in this study have been deposited to the Electron Microscopy Public Image Archive (EMPIAR): BAM-BepA dataset EMPIAR-13664, POTRA-3 crosslink dataset EMPIAR-13669 and barrel crosslink dataset EMPIAR-13670. Details of all deposition codes are also available in Supplementary Table 6. The raw data and all AlphaFold3 predictions are available at the University of Leeds Data Repository (10.5518/1838). Source data are provided in this paper.
