# Supplementary material for: BAM-BepA complexes in outer membrane protein quality control
Source: Nat Commun. 2026 Jul 9;17:8482. doi: 10.1038/s41467-026-75227-x (PMC13478230; doi:10.1038/s41467-026-75227-x)
Supplement: Supplementary file 1 [file 41467_2026_75227_MOESM1_ESM.pdf]

BAM-BepA complexes in outer membrane protein quality control

Katherine L. Fenn, Victoria Higgins, Jonathan M. Machin, Antonio N. Calabrese, Alan Berry, Sheena E. Radford[†], Neil A. Ranson[†]

Supplementary Figures and Supplementary Information

**Supplementary Figure 1**	**Structures of BAM and BepA**
**Supplementary Figure 2**	**CryoEM of BAM-BepA**
**Supplementary Figure 3**	**Interaction between BAM and BepA in the Encounter and Poised complexes**
**Supplementary Figure 4**	**Alphafold3 of BAM-BepA from diderm WHO priority pathogens genera**
**Supplementary Figure 5**	**BepA's lid is dynamic**
**Supplementary Figure 6**	**Engineered disulphide that shuts lid of BepA is buried**
**Supplementary Figure 7**	**CryoEM of BAM-BepA (POTRA-3 crosslink) – Poised Complex**
**Supplementary Figure 8**	**CryoEM of BAM-BepA (barrel crosslink) – Lid-engaged Complex**
**Supplementary Figure 9**	**Interaction between BAM and BepA in the Poised and Lid-engaged complexes**
**Supplementary Figure 10**	**Proteolysis of QMN peptide by BepA in the presence/absence of BAM.**
**Supplementary Figure 11**	**Comparison of the BAM's interaction with BepA and SurA**
**Supplementary Figure 12**	**Positioning of stalled OMPs relative to lid-engaged BepA**
**Supplementary Figure 13**	**Other Enzymes with regulatory histidine in a plug**

**Supplementary Table 1**	**List of Plasmids**
**Supplementary Table 2**	**List of Primers**
**Supplementary Table 3**	**List of Strains**
**Supplementary Table 4**	**List of AF3 Proteins**
**Supplementary Table 5**	**CryoEM Data Collection Parameters**
**Supplementary Table 6**	**Model building parameters**

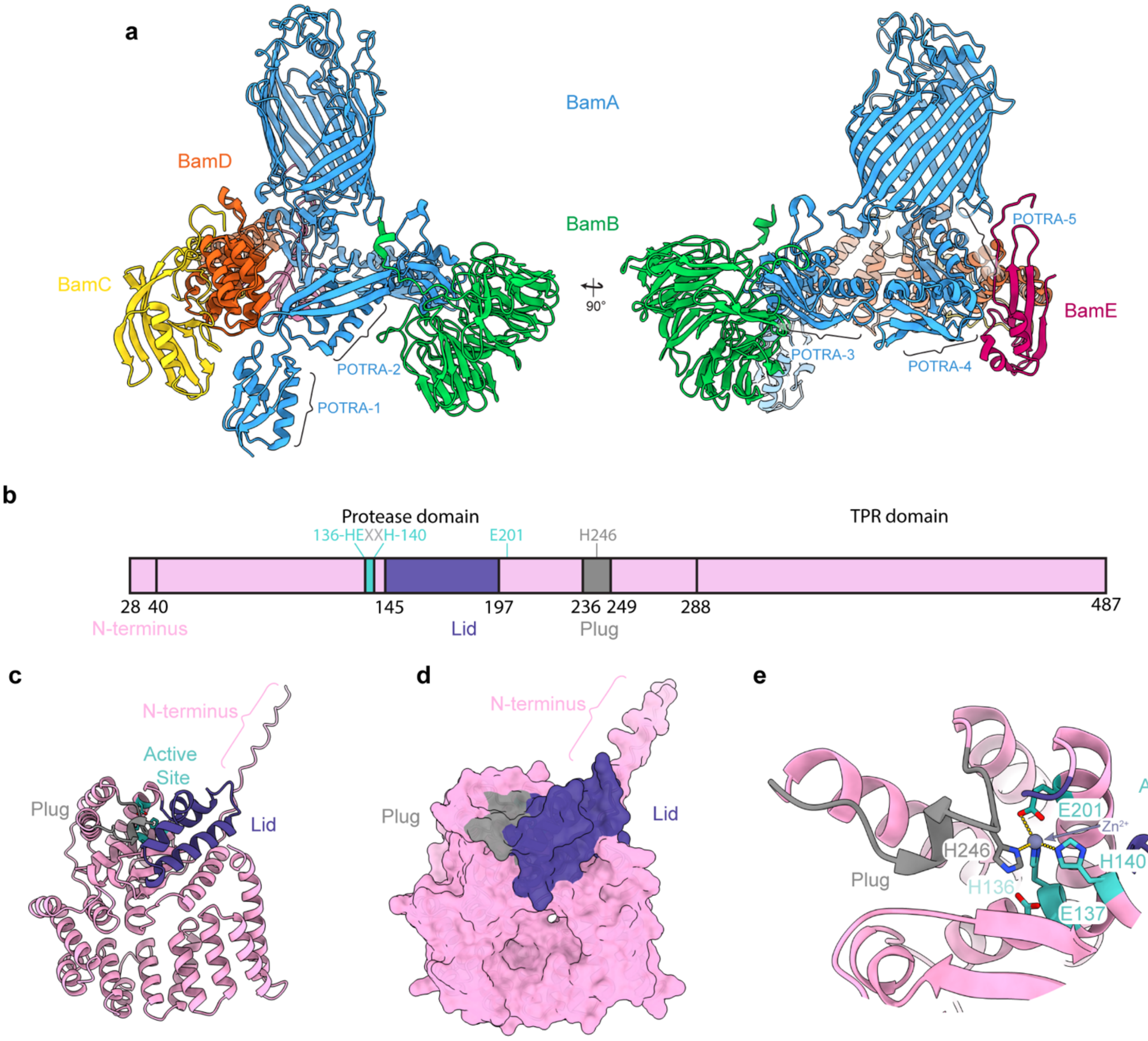

Supplementary Figure 1: Structures of BepA and BAM

a. Structure of BAM in a lateral open conformation (BamABCDE coloured by protein, same colours used throughout). (PDB 5LJO[1], note the second helix grip domain of BamC is not resolved in this structure). **b.** BepA domain/feature organisation. (Note: BepA sequence starts at residue 28 because residues 1-27 are the signal sequence removed by the signal peptidase during secretion into the periplasm.) **c.** Structure of BepA (generated by Alphafold3 (AF3)[2]) in cartoon and **d.** surface representation to demonstrate the occulsion of the active site (teal) by the plug (grey) and lid (dark blue) of BepA. **e.** Active site of BepA in an inactive state showing coordination of the zinc ion by four protein ligands, H136, H140, E201 and H246. 136-HEXXH-140 is a conserved motif and E137 likely acts as the general base to aid deprotonation of a catalytic water molecule in active enzyme conformations[3,4].

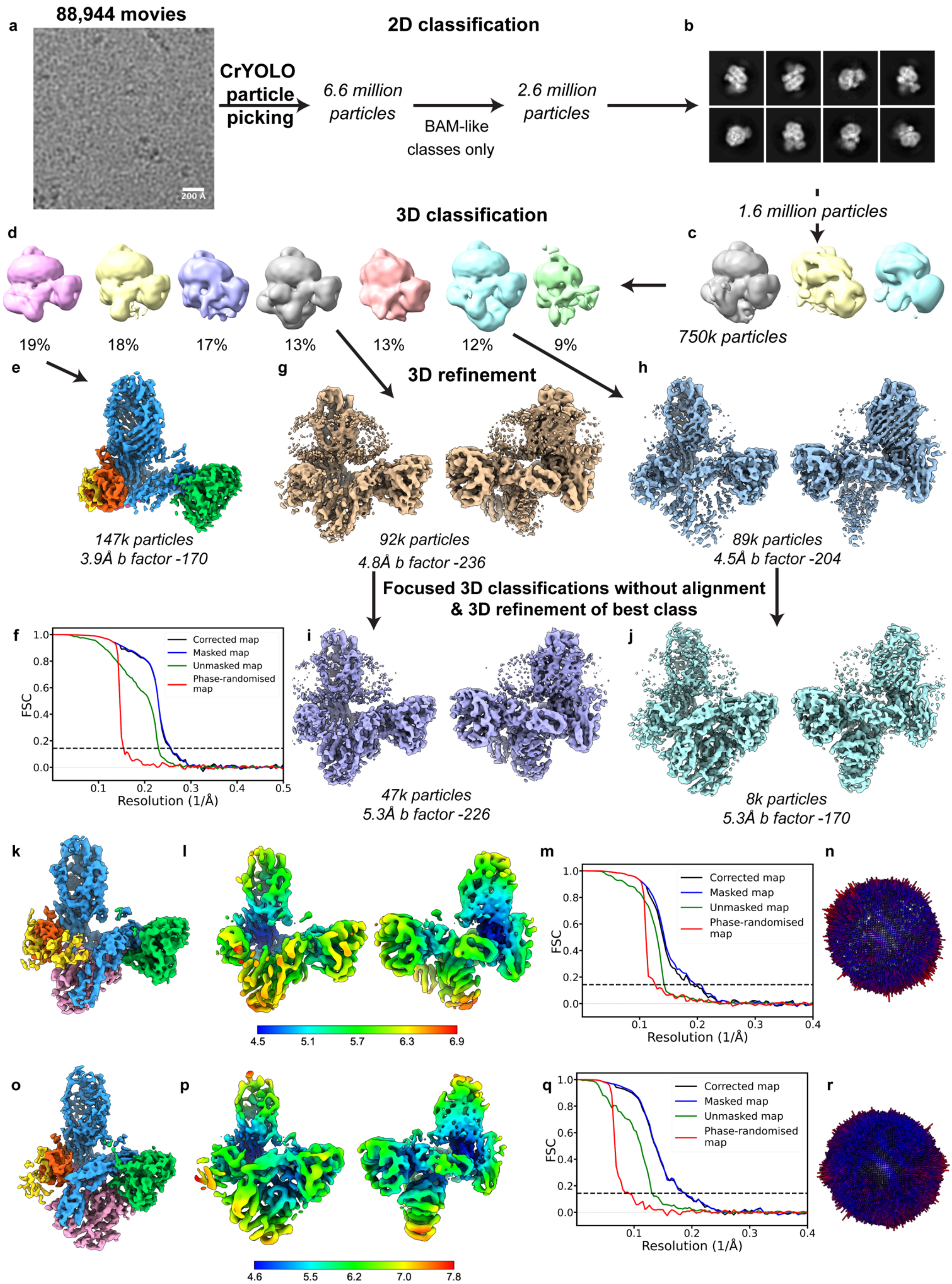

Supplementary Figure 2: CryoEM of BAM-BepA

a. Representative raw electron micrograph and **b.** representative 2D classes. **c.** 3-class 3D classification where the high-resolution class was classified in **a. d.** 7-class 3D classification. **e.** Final reconstruction of apo-BAM after iterative polishing/CTF refinement coloured by BAM subunit. **f.** FSC curve for apo-BAM. **g.** Initial 3D refinement of novel BAM conformation bound BepA and **h.** BAM bound BepA. Both showed poor density for BepA and were focused classified without alignment using masks around the POTRA's and BepA and the best class 3D refined (**i and j**). **k.** Final reconstruction of Poised Complex coloured by protein subunit and **l** by resolution. **m.** FSC curve and **n.** angular distribution. **o.** Final reconstruction of Encounter Complex coloured by protein subunit and **p** by resolution. **q.** FSC curve and **r.** angular distribution.

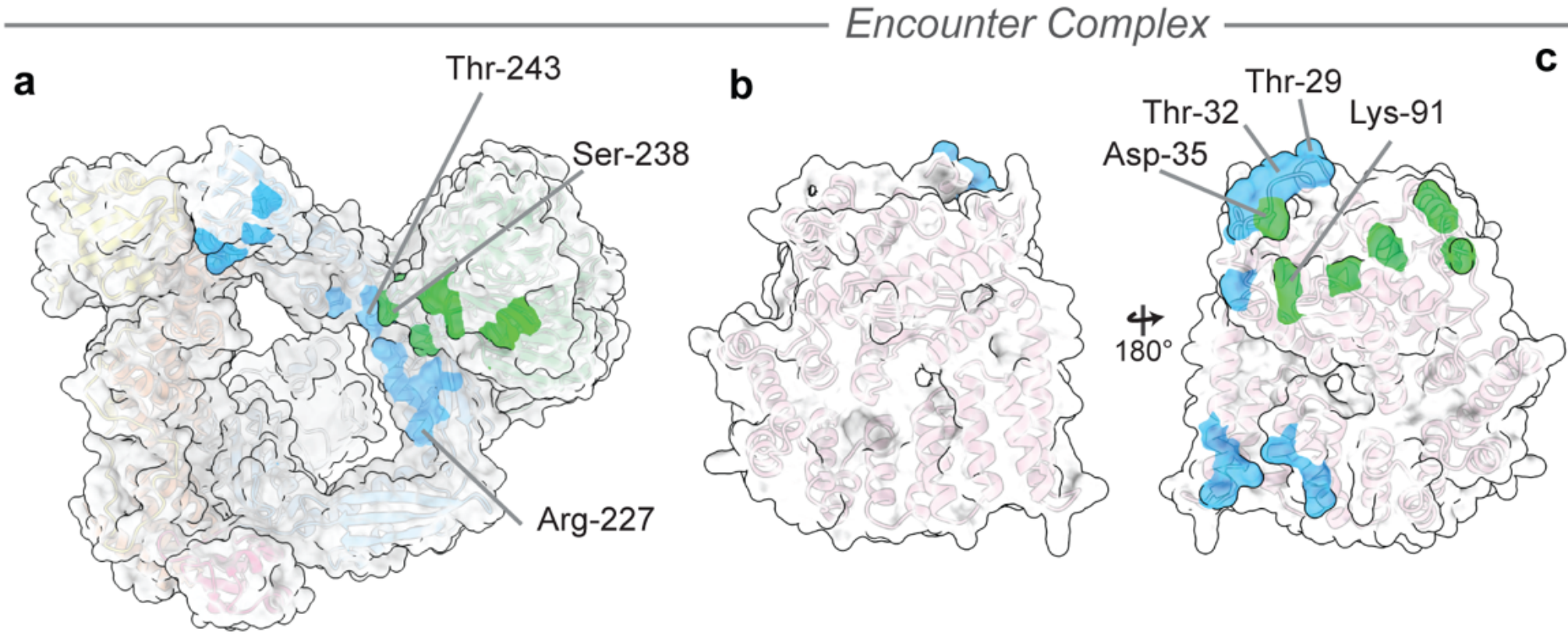

c

BepA	BamA	BamB
Ser-29	Arg-227	
Thr-32	Thr-243	
Asp-35		Ser-238
Lys-91		Ser-238

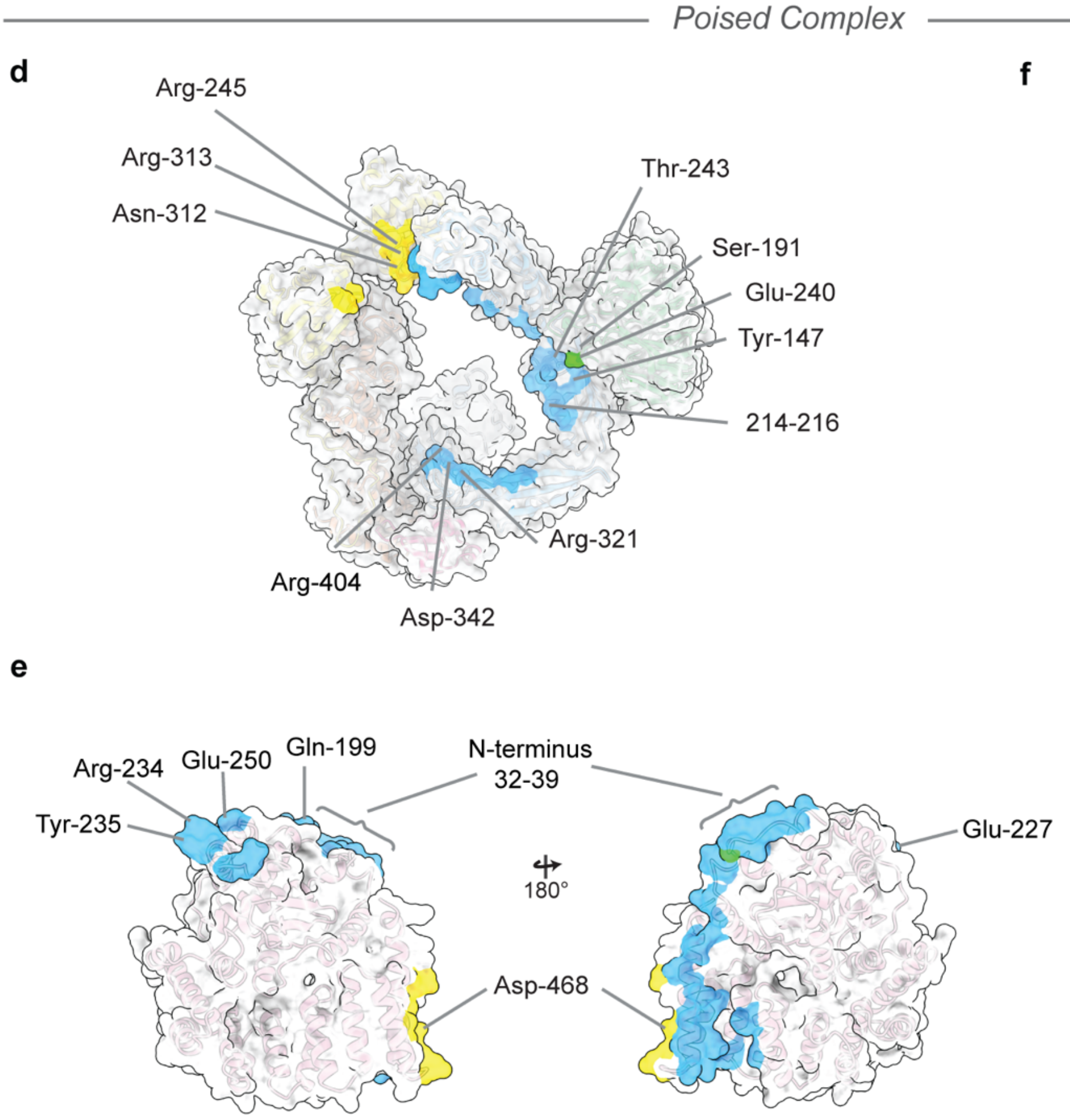

f

BepA	BamA	BamB	BamC
Thr-32	Thr-243		
Thr-32		Ser-191	
Pro-34	Tyr-214		
Asp-35	Lys-216		
Met-36	Tyr-147		
Met-36		Glu-240	
Gly-37		Glu-240	
Ser-39	Gln-215		
Gln-199	Gln-217		
Arg-234	Asp-342		
Glu-227	Arg-321		
Tyr-235	Arg-404		
Ser-236	Arg-404		
Glu-250	Arg-321		
Asp-468			Arg-245
Asp-468			Arg-313
Asp-468			Asn-312

Supplementary Figure 3: Interactions of BAM and BepA in the Encounter and Poised Complexes
a. The BepA interaction interface within the periplasmic ring of the Encounter Complex. BepA interacts with POTRA-1, -2, -3 and BamB with an interaction interface of 766Å^2. (BAM viewed from the periplasmic side upwards towards the barrel with BepA removed for clarity and its interaction interface coloured by protein, **Fig. 1c** shows the same image but without labels). **b.** The BAM interaction sites of BepA in the Encounter complex coloured by BAM subunit. **c.** Table of interacting residues that are labelled in **a.** and **b.** that are suggested to be in close proximity for bonding from our rigid body fit of BepA with the BAM complex. **d.** The BepA interaction interface within the periplasmic ring of the Poised Complex. BepA interacts with POTRA-1,- 2, -3, -4, BamB and BamC with an interaction interface of 1789Å^2. (BAM viewed from the periplasmic side upwards towards the barrel with BepA removed for clarity and its interaction interface coloured by protein, **Fig. 1d** shows the same image but without labels) **e.** The BAM interaction sites of BepA in the Poised complex coloured by BAM subunit. **c.** Table of interacting residues that are labelled in **e.** and **f.**, unlabelled but coloured surfaces indicate van der Waals interactions.

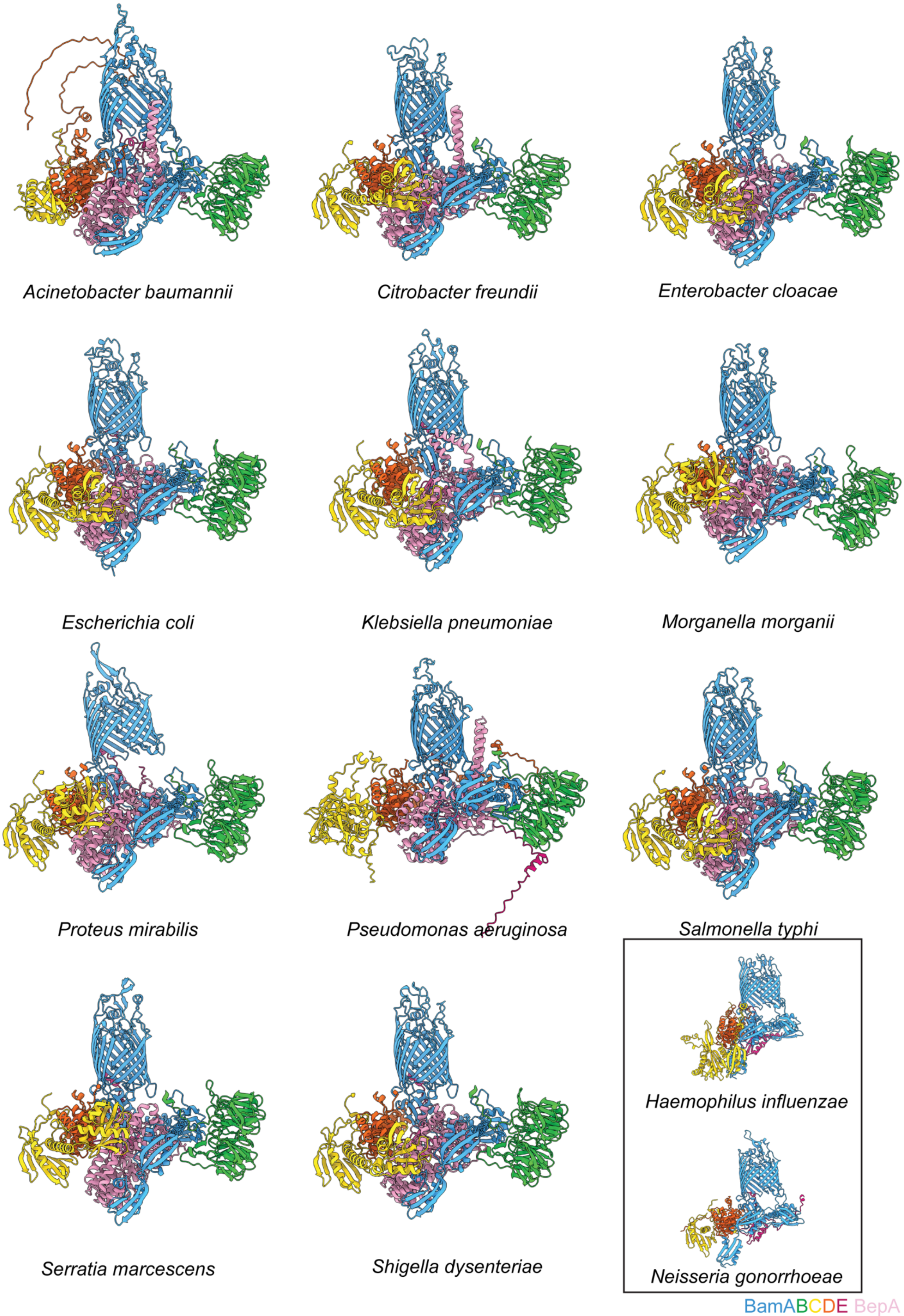

Supplementary Figure 4: Alphafold3 models of BAM-BepA from diderm WHO priority pathogens

Proteins were identified by sequence and structural homology compared to their *E. coli* counterparts. The highest priority species from the WHO priority pathogen list[5] was chosen where multiple genera occurred. Where a specific species was not indicated the most common species was utilised. The individual proteins and the complex architecture is structurally well conserved across all species. The top AF3 model out of five is displayed. The BamA subunit of the 11 complexes has five POTRA domains with the interaction between the POTRA domains, BamB and the BepA N-terminus conserved across all of these complexes. (The list of each protein used is given in **Supplementary Table 4**). *H. influenzae* and *N. gonorrhoeae* have no BamB and also lack BepA.

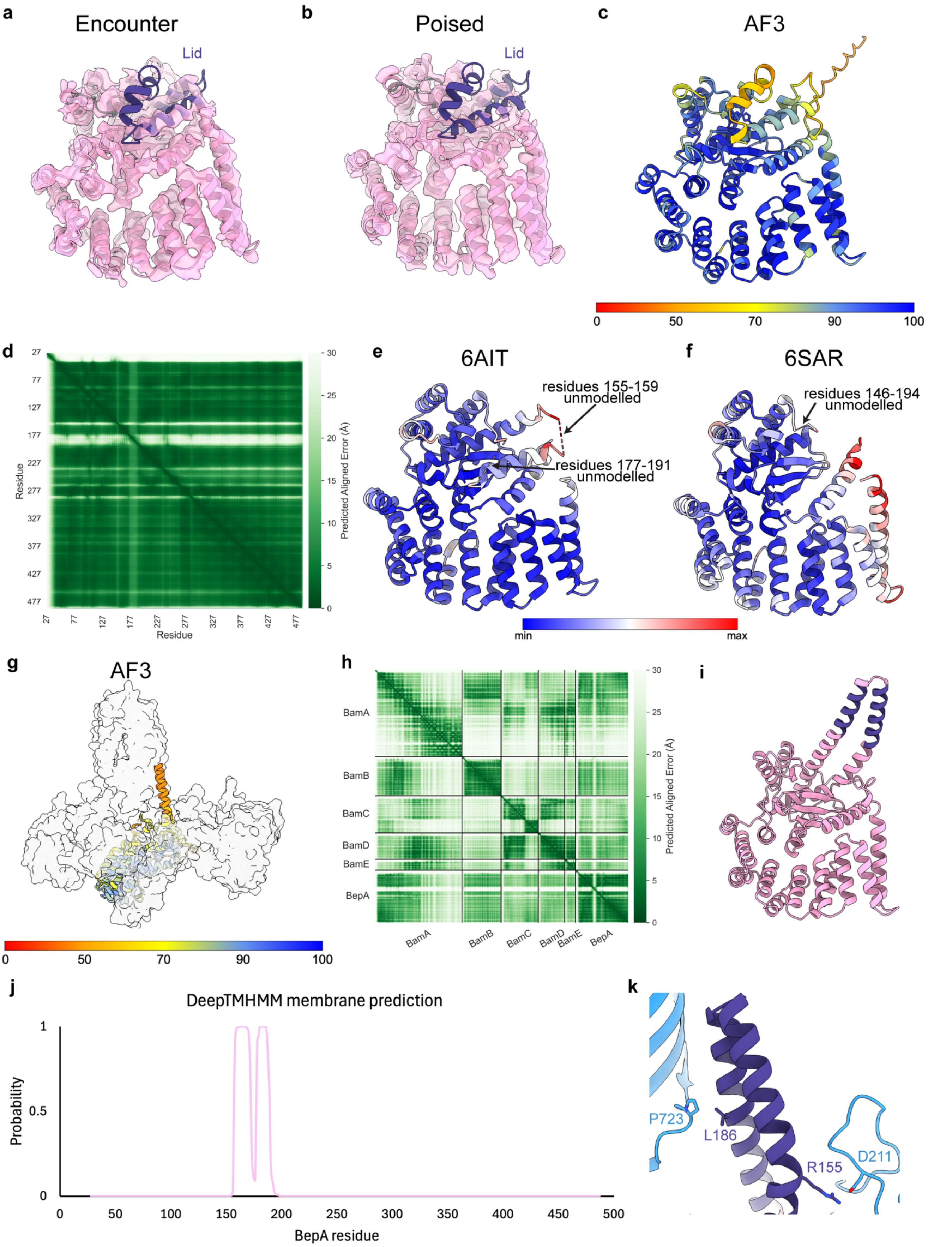

Supplementary Figure 5: BepA's lid is dynamic

CryoEM density for BepA from BAM-BepA structures showing **a.** a partially resolved lid in the Encounter Complex and **b.** an unresolved lid in the Poised Complex. **c.** AF3 model of BepA coloured by plDDT score and **d.** PAE plot **e.** Crystal structure of BepA (PDB 6AIT)[3] coloured by b-factor in which the lid is only partly resolved (residues 155-159 and 177-191 are not modelled). Note protein was expressed without the N-terminus (residues 28-44). **f.** Crystal structure of BepA (PDB 6SAR)[4] coloured by b-factor in which the N-terminal residues 28-43 and residues 146-194 in the lid region are not modelled. **g.** AF3 model of BAM-BepA with lid engaged with BepA extending next to the BamA barrel where BAM is portrayed in grey transparent surface and BepA is coloured by plDDT score and **h.** PAE plot. **i.** AF3 prediction of 'Lid-off' BepA with transmembrane residues coloured purple (residues with probability of membrane burial >0.5 as predicted by DeepTMHMM[6] are coloured purple). **j.** DeepTMHMM[6] membrane prediction of the sequence of BepA. **k.** Residues from AF3 prediction that were mutated to cysteine for lid engagement.

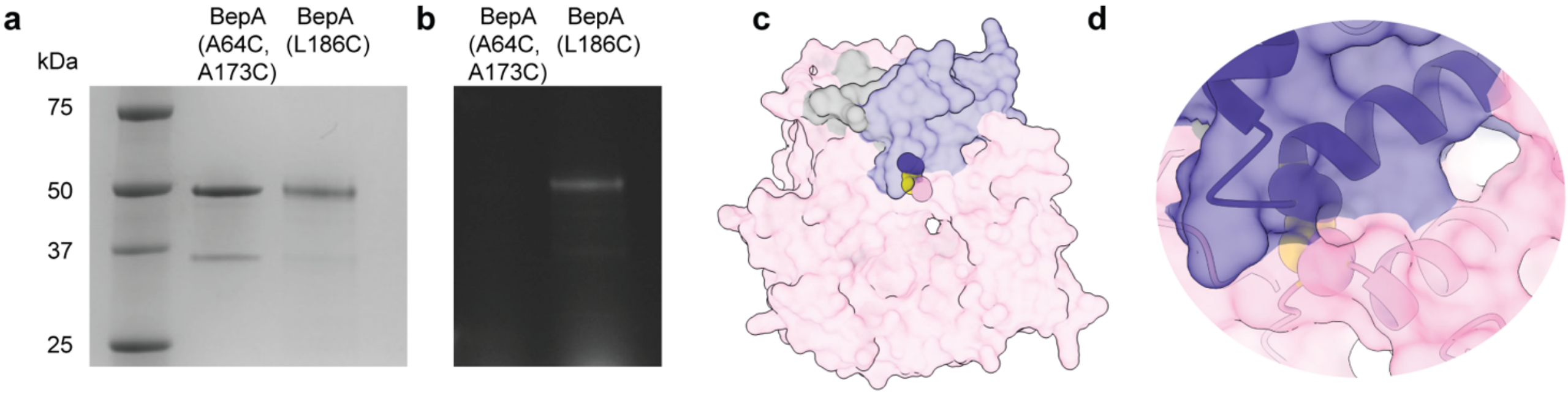

Supplementary Figure 6: Engineered disulphide that shuts lid of BepA is buried.
Purified concentrated samples of lid shut BepA (BepA with double cysteine mutations A64C, A173C) and single cysteine mutant (BepA L186C) were incubated for 30 mins with 50x molar excess of I-aedans reagent to label free cysteines prior to quenching with 10mM DTT. SDS-PAGE gel of both samples **a.** Coomassie stained and **b.** UV imaged. **c. & d.** Location of disulphide bond (A64C,A173C) in Lid Shut BepA coloured yellow with surface accessibility values of 14 and 0Å^2.

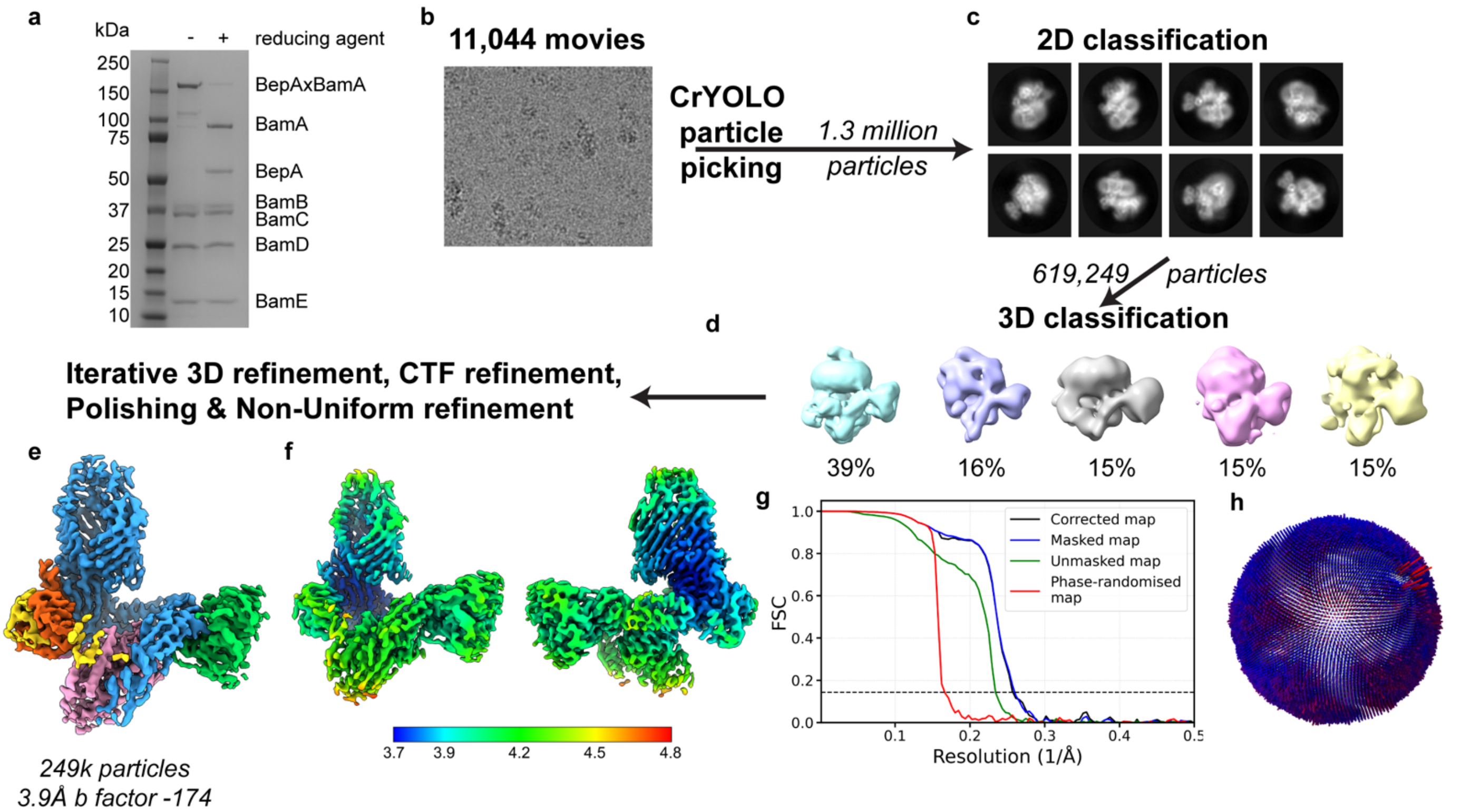

Supplementary Figure 7: CryoEM of BAM-BepA (POTRA-3 disulphide bonded between BepA (R155C) POTRA-3 (D211C)) – Poised Complex

a. SDS PAGE gel of purified concentrated sample. **b.** Representative raw electron micrograph and **c.** representative 2D classes. **d.** 5-class 3D classification where the high-resolution class was selected for iterative 3D refinement, CTF refinement, polishing and non-uniform refinement. **e.** Final reconstruction of Poised Complex coloured by protein **f.** and by local resolution. **g.** FSC curve and **h.** angular distribution.

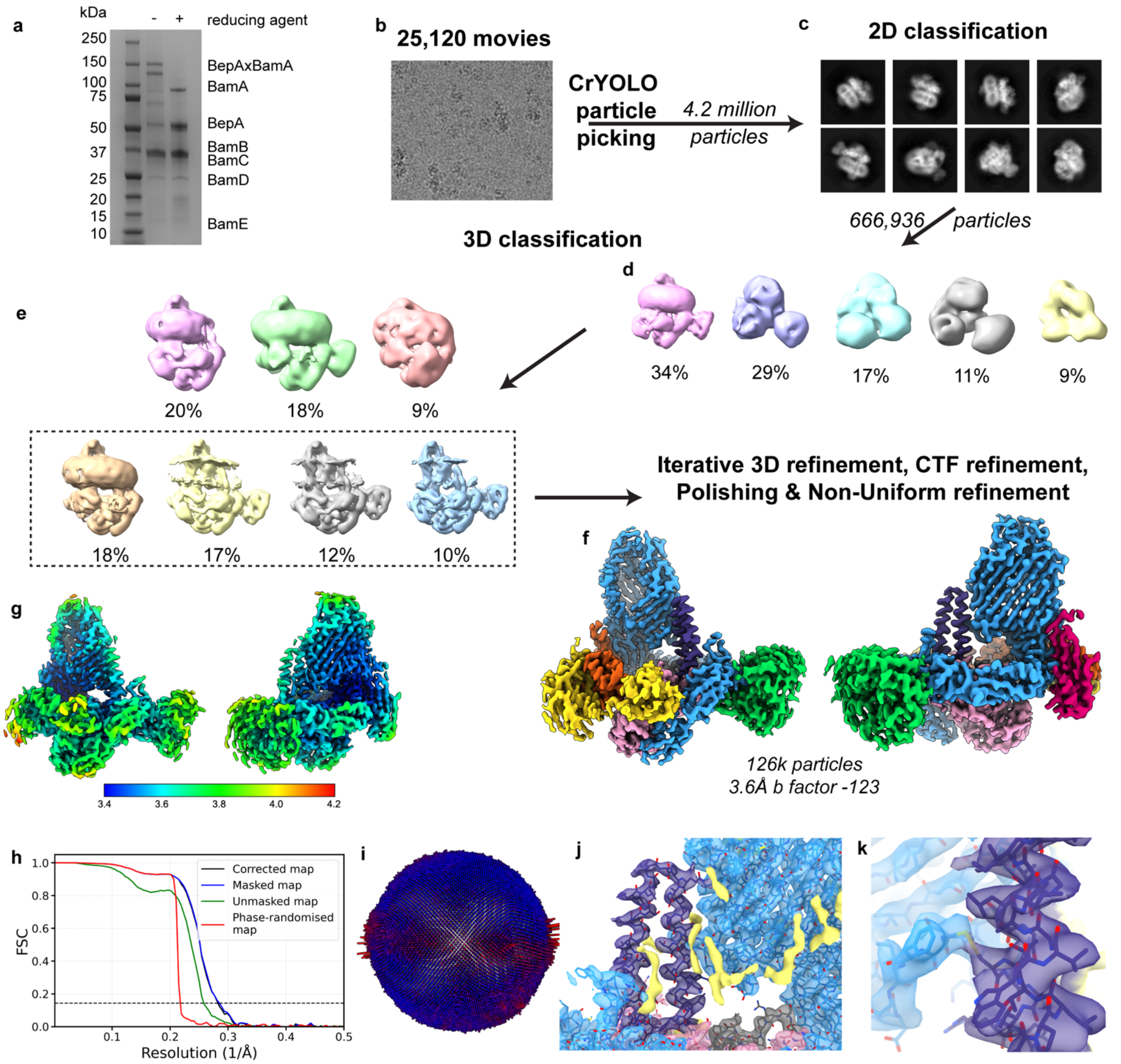

Supplementary Figure 8: CryoEM of BAM-BepA (disulphide between BepA lid (L186C) and turn-6 of the BamA β-barrel (P723C)) – Lid-engaged Complex

a. SDS PAGE gel of purified concentrated sample. **b.** Representative raw electron micrograph and **c.** representative 2D classes. **d.** 5-class 3D classification where the high-resolution class was selected for **e.** 7-class 3D classification. Classes in the box that were the highest resolution were taken forward for iterative 3D refinement, CTF refinement, polishing and non-uniform refinement. **f.** Final reconstruction of Lid-engaged Complex coloured by protein. **g.** and by local resolution **h.** FSC curve and **i.** angular distribution **j.** Map and model of the lid and its interactions with BamA and detergent (yellow density). **k.** Density for disulphide bond formed between the lid and turn-6 of the barrel.

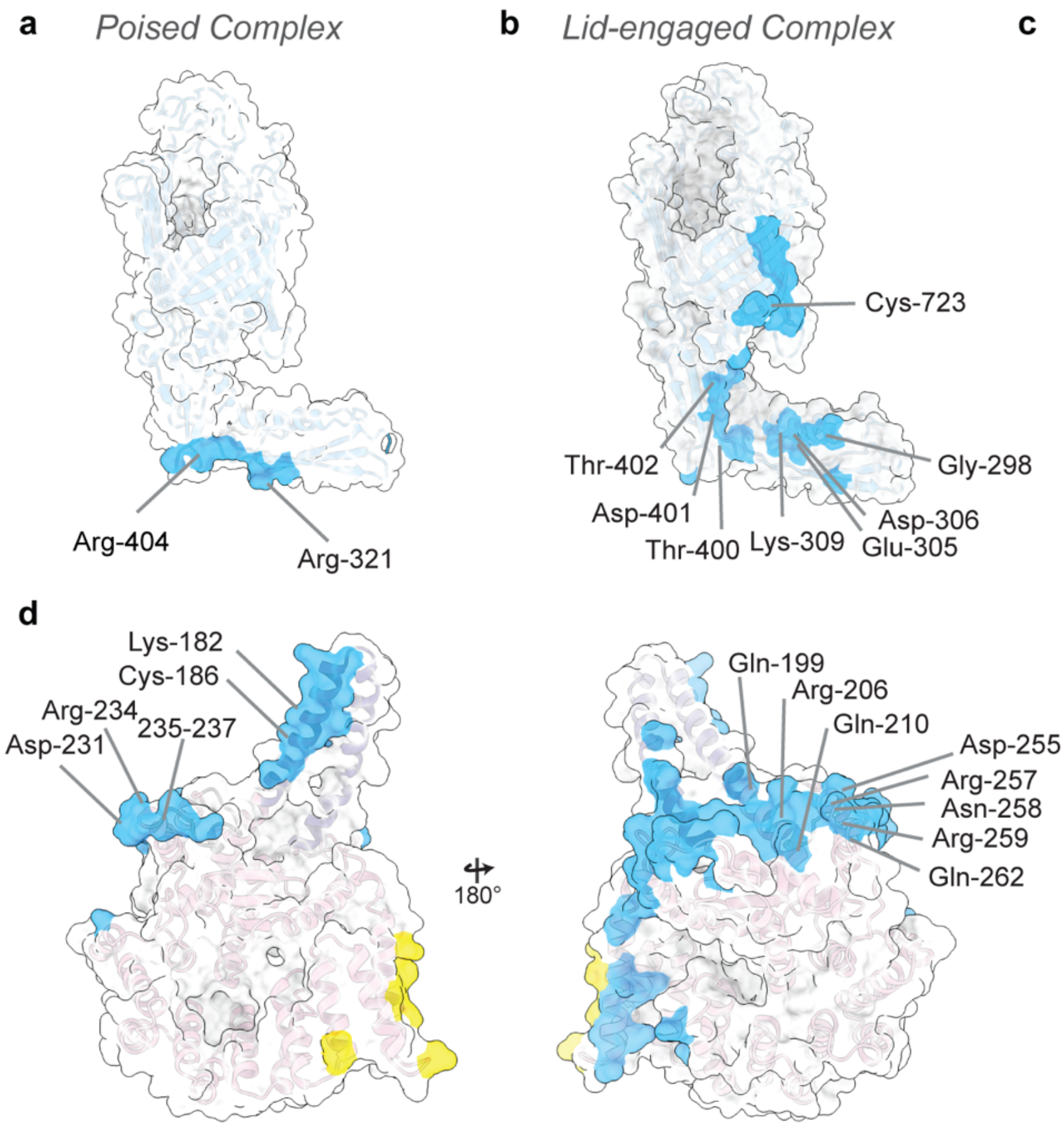

c

BepA	BamA
Asp-31	Lys-216
Asp-31	Lys-216
Pro-34	Tyr-214
Ser-39	Gln-217
Ser-42	Ser-143
Ser-45	Asn-113
Ser-89	Lys-216
Arg-155	Asp-211
Arg-155	Asp-211
Leu-182	Cys-723
Cys-186	Cys-723
Cys-186	Cys-723
Gln-199	Gln-198
Gln-199	Gln-198
Arg-206	Thr-225
Gln-210	Glu-224
Asp-231	Lys-309
Asp-231	Lys-309
Arg-234	Asp-306
Arg-234	Asp-306

BepA	BamA
Tyr-235	Thr-402
Tyr-235	Ala-318
Ser-236	Thr-400
Ser-236	Asp-401
Ser-237	Lys-386
Ser-237	Thr-400
Asp-255	Ser-228
Arg-257	Glu-305
Arg-257	Glu-305
Asn-258	Gly298
Arg-259	Glu-224
Arg-259	Glu-224
Arg-259	Ser-228
Gln-262	Glu-224
Gln-262	Ala-236
Gln-262	Phe-238
Asn-400	Arg-49

Hydrogen Bond
Salt Bridge
Disulphide

Supplementary Figure 9: Interactions of BAM and BepA in Poised Complex and Lid-engaged Complex
a. The BepA interaction interface with POTRA-4 and POTRA-5 of BamA of the poised complex, (**Fig. 3e** shows the same image but without labels). **b.** The BepA interaction interface with POTRA-4, POTRA-5 and the barrel of BamA in the Lid-engaged complex (**Fig. 3f** shows the same image but without labels)**.** (**a.** and **b.** POTRA-4, POTRA-5 and the BamA barrel displayed only). **c.** Table of interacting residues of BamA and BepA in the Lid-engaged complex. New interactions found only in the Lid-engaged complex are labelled in **b.** and **d.**, unlabelled but coloured surfaces indicate van der Waals interactions. **d.** The BAM interaction sites of BepA in the Lid-engaged complex coloured by BAM subunit.

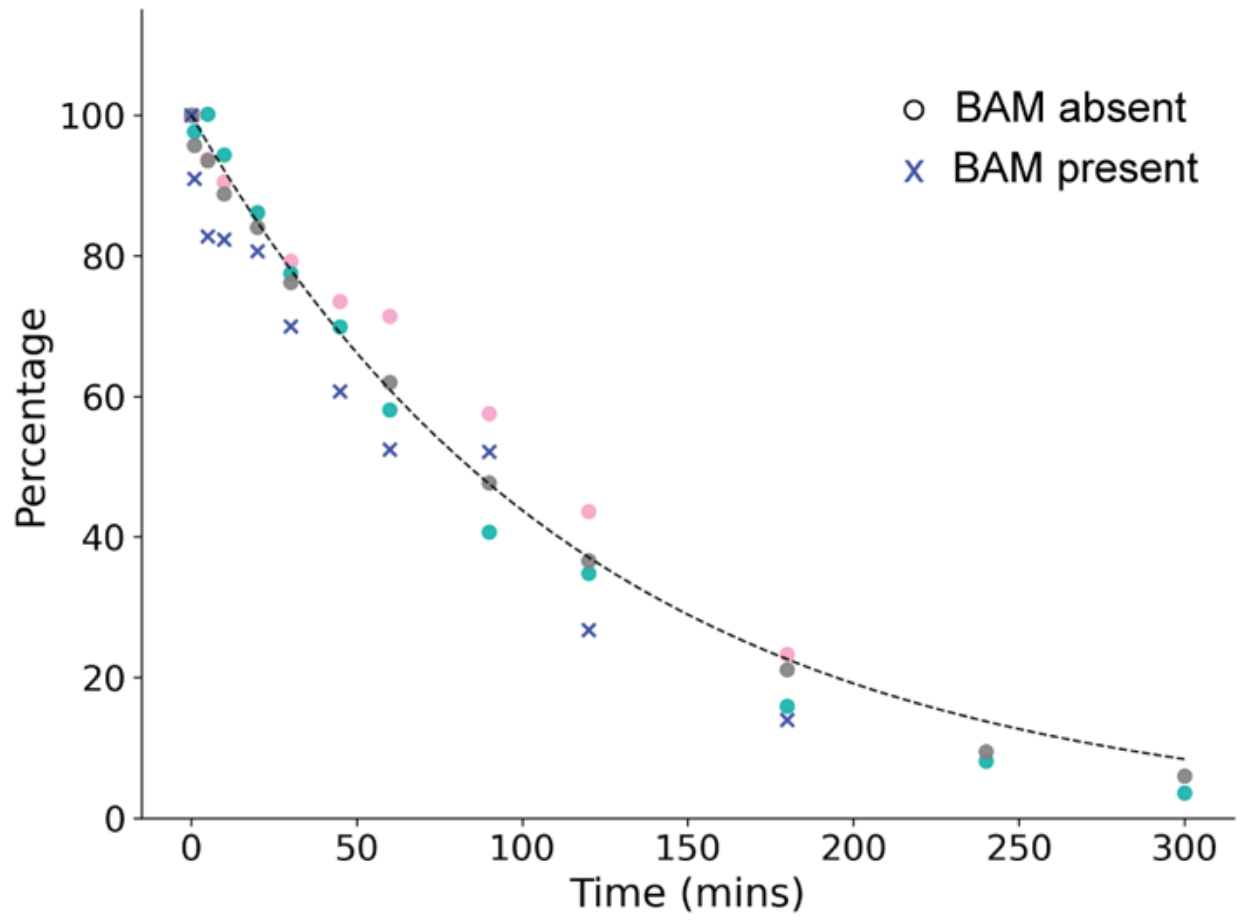

Supplementary Figure 10: Proteolysis of QMN peptide by BepA in the presence/absence of BAM.
BepA mediated degradation of QMN peptide (QMNKMGGFNLKYRYE) over time. Data same as in Figure 5a but including data for proteolysis of the same peptide in the presence of a 14-times molar excess of BAM (42 μM) to BepA plotted (x) (n=1). Data are fitted to the same exponential as in Figure 5a with triplicate data shown.

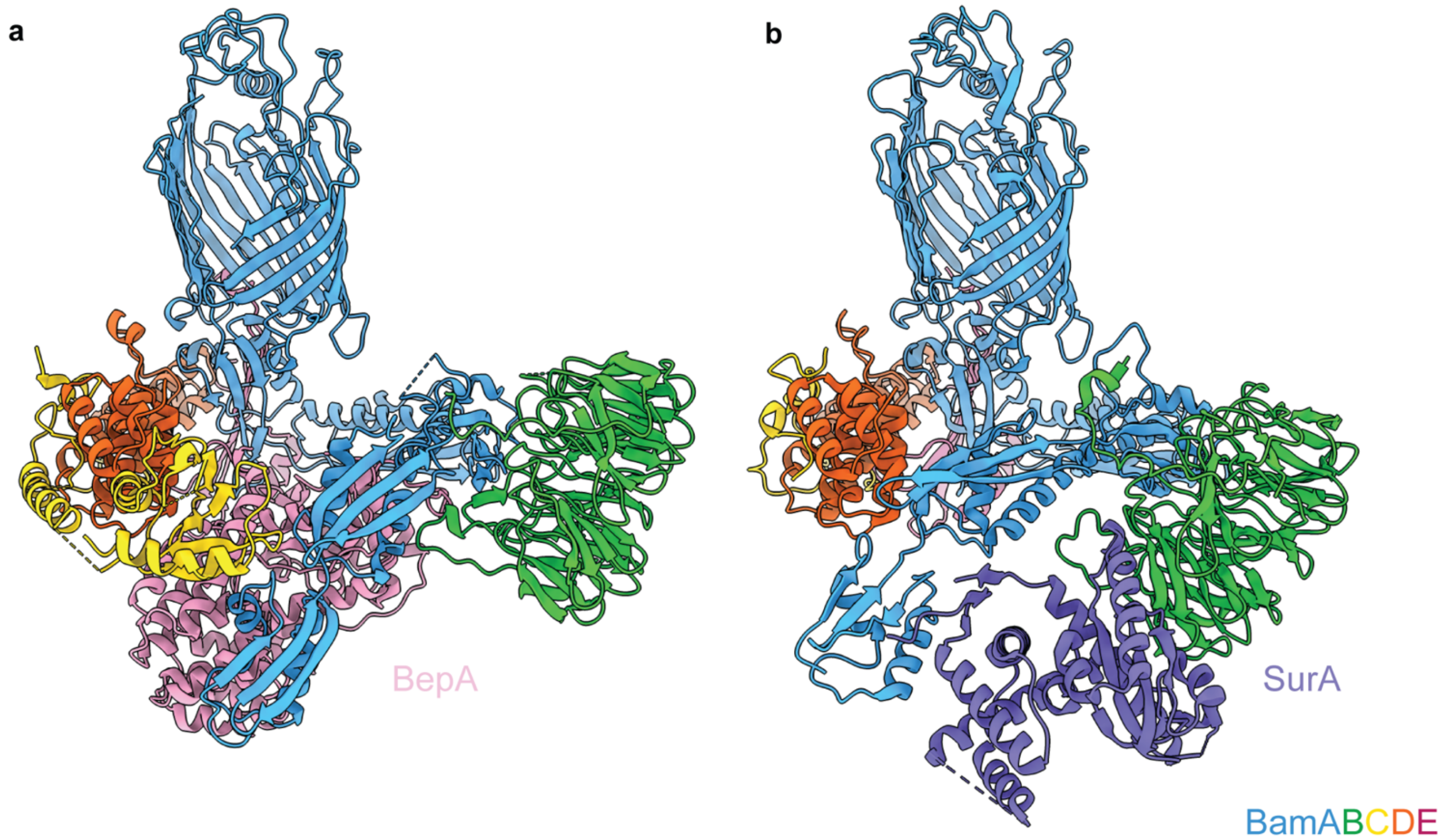

Supplementary Figure 11: Comparison of the BAM's interaction with BepA and SurA.
a. BAM-BepA structure (Poised Complex) **b.** BAM-SurA structure (PDB: 8PZ1)

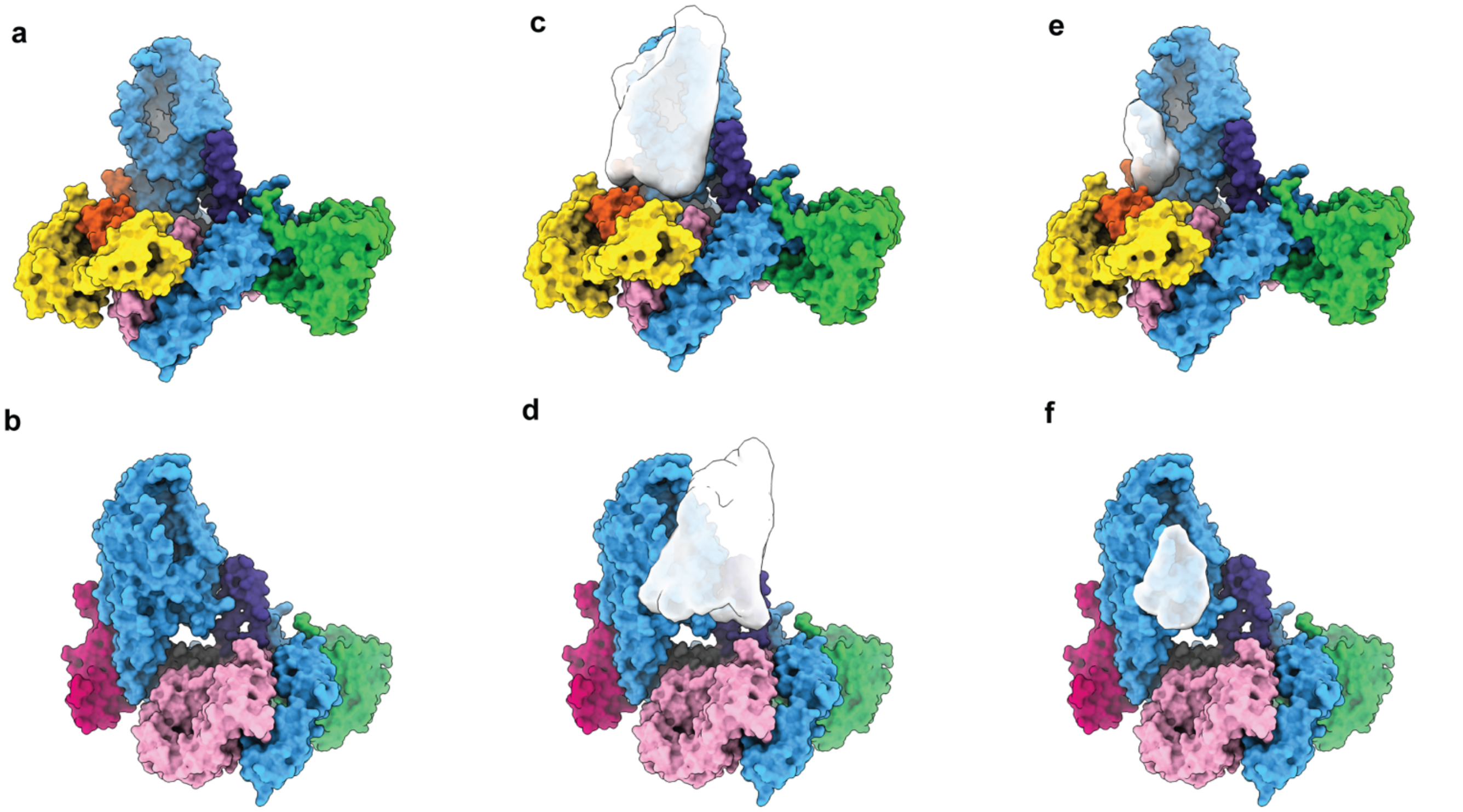

Supplementary Figure 12: Positioning of stalled OMPs relative to lid-engaged BepA

a. Lid-engaged complex showing all subunits **b.** and with BamC and BamD removed to see the active site. **c.** Lid-engaged complex with a late-stage stalled OMP (EspP from 8qp5) shown as molmap representation in transparant white (model was aligned with BamA and then only the OMP shown). **d.** and viewed without BamC and BamD. **e.** Lid-engaged BepA with an early-stage stalled OMP (EspP from 7TTC) shown as molmap respresentation in transparant white (model was aligned with BamA and then only the OMP shown**) f.** viewed without BamC and BamD. (NOTE: **c-f** are alignment's only to aid visulation of position of BepA relative to published stalled OMP structures, it is unknown whether these conformations exist).

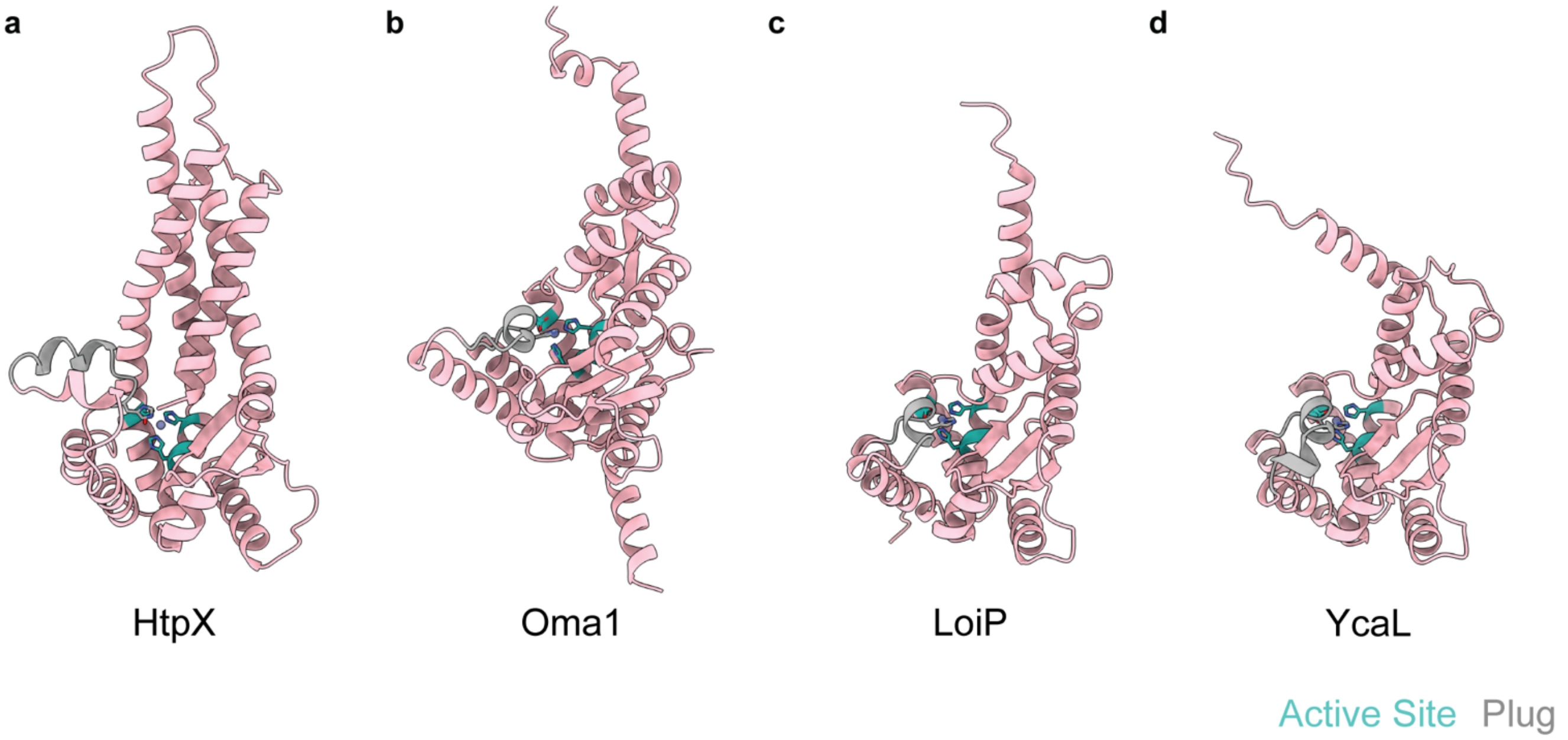

Supplementary Figure 13: Other OM proteases with a regulatory histidine in a plug

AF3 models of **a.** *E. coli* HtpX (Uniprot: p23894), **b.** *S. cerevisiae* mitochondrial Oma1 (Uniprot: p36163), **c.** *E. coli* LoiP (Uniprot: P25894), **d.** *E. coli* YcaL (Uniprot: p43674). All predictions with a Zn^{2+} ion.

Supplementary Table 1: List of Plasmids

Plasmid Name	Usage	Source
pSCRhaB2-BepA	Expression	This study
pSCRhaB2-BepA-CTTS	Expression	This study
pSCRhaB2-BepA(A64C, A173C)-CTTS	Expression	This study
pSCRhaB2-BepA(E103C, E241C)-CTTS	Expression	This study
pSCRhaB2-BepA(R155C)-CTTS	Expression	This study
pSCRhaB2-BepA(R190C)-CTTS	Expression	This study
pTrc99a2-BamABCDE-CT8His	Expression	Roman-Hernandez et al. 2014
pTrc99a2-BamA(D211C)BCDE-CT8His	Expression	This study
pTrc99a2-BamA(P723C)BCDE-CT8His	Expression	This study
pZA31-BepA	Complementation	This study
pZA31-BepA(Δ28-40)	Complementation	This study
pZA31-BepA (A64C,A173C)	Complementation	This study

Supplementary Table 2: List of Primers

Primer Description	Forward sequence	Reverse Sequence
Amplification of BepA plus RBS from E. coli BW25113 genome with overhang ends of NdeI and HindIII cut sites	TAAGCACATATGATCTCCAGAAATACA GGATAGAGGT	TAAGCAAAGCTTGTCATTGTTCTTCCTTT AATGCGAA
Insert of a GSS followed by a TwinStrep tag to the C-terminus of pSCRhaB2-BepA to create pSCRhaB2-BepA-CTTS	GGTGGTGGTTCTGGCGGATCGGCCTG GTCACACCCCCAATTCGAGAAGTAAAA GCTTGGCTGTTTTG	TGATCCGCCACCCTTCTCGAATTGTGG GTGAGACCAGGCGCTGCTGCCCATCTT GGTATAAGGCTTAAAG
Insertion of BepA into pZA31 by Gibson assembly	ATGGGCAGCAGCTAGGGTATCGATAA GCTTG	CCTGAACATGTCGACCTCGAGG
linearisation of pZA31 for addition of BepA by Gibson assembly	GAGGTCGACATGTTCAGGCAGTTGA	TATCGATACCCTAGCTGCTGCCCATCTT G
Deletion of residues 28-40 from BepA sequence	GGAAGCACGCTTTCCATTGGTC	GGCAAACGCCGGGGCTAC
Mutation A64C in BepA sequence	ACGCGGCAGCTGCCCGTTAATTAATG	AGCTGGCGGACATAATAG
Mutation A173C in BepA sequence	ACTGGCGATGTGCAGTCCGCAGG	AAAATAGAACCTAACGCGC
Mutation of R155C in BepA sequence	AGATCAGCAGTGCAGCGCGCCGC	AGATCAGCAGTGCAGCGCGCCGC
Mutation of R190C in BepA sequence	GGCGGGAACGTGCCAGGGGATGA	AGTGTACCGGTCAGCGCC
Mutation of E103C in BepA sequence	CAACAACGACTGCATTAACGCCTTTG	ATCAGAAAAAAATGAAACGG
Mutation of E241C in BepA sequence	GCGCCCGCCGTGCATTTTATTGAC	GAGGAGTAACGCGCC
Mutation of D211C in BamA sequence	CGTGGTAGGCTGCCGTAAATACCAGA AACAG	TTCCACCACGGCACTTCG
Mutation of P723C in BamA sequence	CACCCCGACGTGCTTTATTAGCGATAA GTATGCTAAC	ATGAACTCGAGGCTGGCA

Supplementary Table 3: List of Strains

Strain Name	Genotype	Source
BL21 (DE3)	*F–ompT hsdSB (rB–, mB–) gal dcm (DE3)*	NEB
BW25113 ΔBepA	Δ(araD-araB)567 ΔlacZ4787(::rrnB-3) λ- rph-1 Δ(rhaD-rhaB)568 hsdR514 ΔbepA	Horizon Discovery, Keio Knockout Collection

Supplementary Table 4: List of AF3 Proteins

Species	BamA	BamB	BamC	BamD	BamE	BepA
Acinetobacter baumannii	A0A7S8WB78	A0A6F8TKL1	A0AA45B997	A0A1E3M1K3	A0A0M3FHZ0	0A385EXL8
Citrobacter freundii	A0A859T7H0	A0A7G2IZA3	A0A859TJK4	A0A7D6Z648	A0A7G2IYJ2	A0A7W3E5S3
Enterobacter cloacae	A0A6S5JMV2	A0A6S5JQM1	A0A6S5K0U2	A0A7H8UEA5	A0A0X4AWL0	A0A7H8UFM0
Escherichia coli	P0A940	P77774	P0A903	P0AC02	P0A937	P66948
Haemophilus influenzae	P46024	-	A0AAE8D2C4	A0A2S9RQB9	A0A2S9S3M2	-
Klebsiella pneumoniae	A0A8D6Q4R0	A0A486R062	A0A7X1HU83	A0A9Q4RR01	A0A5D3JF05	A0A483FPG
Morganella morganii	J7TNX3	J7U6S6	J7TFW8	M1SDT4	M1SEW5	J7U3S3
Neisseria gonorrhoeae	P95359	-	A0A1D3J262	Q50985	A0A1D3EW25	-
Pseudomonas aeruginosa	A0A232C799	A0A3S0IXX2	A0A3S0LDF5	A0A509JJN6	A0A9Q9K074	A0A069QD99
Proteus mirabilis	A0A1Z1SSX0	A0A1Z1STA0	A0A1Z1SUX5	A0A1Z1SXR8	A0A1Z1ST44	A0A2X2BCU1
Salmonella typhi	Q8Z9A3	A0A716WIB5	A0A748ESA5	A0A3Z6T0V7	A0A751N435	P66951
Serratia marcescens	A0A2S4X5B9	A0A2V4FU69	A0A2S4X671	A0A086FBJ7	A0A084X0E5	A0A2V4G067
Shigella dysenteriae	A0A3R0X394	A0A403M3S6	A0A403M4Y4	A0A403M6N3	A0A2S8E8A5	A0A2X2I658

Supplementary Table 5: CryoEM Data Collection Parameters

Dataset	BAM BepA Collection 1	BAM BepA Collection 2	BAMxBepA (POTRA-3 Crosslink)	BAMxBepA (Barrel Crosslink)
Magnification	165k	165k	165k	165k
Pixel Size (Å)	0.74	0.74	0.76	0.76
Micrographs	28,320	60,624	11,044	25,120
Dose per pixel/second	7.73	7.35	8.55	7.49
Dpse per Å^2/second	14.11	13.42	15.6	12.96
Exposure time	2.87	3.4	2.61	3.07
Total Dose	40.5	45.6	40.8	39.8
Dose per frame	1.23	1.17	1.36	1.14
Frames	33	39	30	35
EMPIAR	EMPIAR-13664 Grid1-2	Grid3-6	EMPIAR-13669	EMPIAR-13670

Supplementary Table 6: Modelling Statistics Table

**Dataset**	BAM BepA		BAMxBepA (POTRA-3 crosslink)	BAMxBepA (Barrel crosslink)
**Structure**	Encounter Complex	Poised Complex	Poised Complex	Lid-engaged Complex
**EMDB Code**	EMD-56550	EMD-56559	EMD-56549	EMD-56543
**PDB Code**	28JR	28JU	28JQ	28JL
**Map**				
**Pixel size (Å)**	0.74	0.74	0.74	0.76
**Resolution (Å) (0.143 FSC threshold)**	5.3	5.3	3.9	3.6
**Sharpening *B* factor (Å^2)**	-170	-226	-174	-123
**Model Refinement**				
**Initial model (PDB)**				
BAM	5LJO	28JQ	5LJO	5LJO
BepA	AF3 lid-on	28JQ	AF3 lid-on	AF3 lid-off
**Model resolution (Å) (0.5 FSC threshold)**			3.9	3.7
**Model map correlation**			0.81	0.79
**Model composition**				
Non-hydrogen atoms			15715	17106
Protein residues			2036	2226
Ligands			1	1
**Protein B factors (Å^2)**			69.19	171.2
**R.M.S. deviations**				
Bond lengths (Å)	N/A – rigid body fit		0.005	0.003
Bond angles (°)			0.645	1.019
**Validation**				
MolProbity score			2.58	1.85
Clashscore			11.98	3.79
Poor rotamers (%)			5.89	3.25
**Ramachandran plot**				
Favoured (%)			93.97	95.70
Allowed (%)			6.03	3.85
Disallowed (%)			0	0.45

References

1. Iadanza, M. G. *et al.* Lateral opening in the intact β-barrel assembly machinery captured by cryo-EM. *Nat. Commun.* **7**, 12865 (2016).

2. Abramson, J. *et al.* Accurate structure prediction of biomolecular interactions with AlphaFold 3. *Nature 2024 630:8016* **630**, 493–500 (2024).

3. Shahrizal, M. *et al.* Structural Basis for the Function of the β-Barrel Assembly-Enhancing Protease BepA. *J. Mol. Biol.* **431**, 625–635 (2019).

4. Bryant, J. A. *et al.* Structure-function characterization of the conserved regulatory mechanism of the Escherichia coli M48 metalloprotease BepA. *J. Bacteriol.* **203**, e00434-20 (2021).

5. World Health Organization. WHO Bacterial Priority Pathogens List, 2024- Bacterial pathogens of public health importance to guide research, development and strategies to prevent and control antimicrobial resistance. *World Health Organization* 1–72 (2024).

6. Hallgren, J. *et al.* DeepTMHMM predicts alpha and beta transmembrane proteins using deep neural networks. BioRXiv (2022) doi:10.1101/2022.04.08.487609.
