# Supplementary material for: BAM-BepA complexes in outer membrane protein quality control
Source: Nat Commun. 2026 Jul 9;17:8482. doi: 10.1038/s41467-026-75227-x (PMC13478230; doi:10.1038/s41467-026-75227-x)
Supplement: Supplementary file 2 [file 41467_2026_75227_MOESM2_ESM.pdf]

Description of Additional Supplementary Files

File name: Supplementary Movie 1
Description: Morph between Encounter and Poised Complex Morphing trajectory between Encounter and Poised Complex generated in UCSF Chimera. Intermediate frames were computed by linear interpolation of atomic coordinates to illustrate the conformational transition. The lid was modelled in using the apo-BepA AF3 model.

File name: Supplementary Movie 2
Description: Morph between Poised and Lid-engaged Complex Morphing trajectory between Poised and Lid-engaged Complex generated in UCSF Chimera. Intermediate frames were computed by linear interpolation of atomic coordinates to illustrate the conformational transition. The lid was modelled into the Poised Complex using the apo-BepA AF3 model.
