# Supplementary material for: BAM-BepA complexes in outer membrane protein quality control
Source: Nat Commun. 2026 Jul 9;17:8482. doi: 10.1038/s41467-026-75227-x (PMC13478230; doi:10.1038/s41467-026-75227-x)
Supplement: Supplementary file 4 [file 41467_2026_75227_MOESM6_ESM.pdf]

nature portfolio

Peer Review File

BAM-BepA complexes in outer membrane protein quality control

Corresponding Author: Professor Neil Ranson

This file contains all reviewer reports in order by version, followed by all author rebuttals in order by version.

Version 0:

Reviewer comments:

Reviewer #1

(Remarks to the Author)
Protein degradation at the bacterial outer membrane remains very poorly understood but one of the key proteases that have emerged in recent years is BepA. The importance of BepA is clear given the antibiotic permeability defects observed for a bepA null mutant. Here Fenn et al use cryoEM, AF3 predictions, and proteolysis assays to finely characterise previously unknown states of BepA as it is bound to BAM revealing a plausible path of conformational changes to bring the BepA active pocket in close proximity to where a new OMP would be folding into the membrane while bound to BamA in a hybrid-barrel conformation. This is excellent work and I would encourage publication in Nature Communications. I have only one minor comment potentially worth addressing with an additional experiment (1) and several other minor comments:

(1) In Figure 2c the lid shut vancomycin sensitivity phenotype is interesting but the result would be much more meaningful if the level of disulfide formation was also determined. Is the phenotype resultant of 90% of BepA forming a disulfide between A64C-A173C or did only a small fraction of BepA oxidise? It might be nice to blot lysates to detect a BepA band shift that would correspond with the level of disulfide oxidation. Also, it would be helpful to readers to show on a model placed into the figure where the cysteines were engineered.

(2) To trap the lid engaged complex the authors engineered intermolecular disulfides: BepA(R155C)-BamA(D211C) [moderate success], and BepA(L186C)-BamA(P723C) [high success]. It would be very helpful to add a model to the figure with these positions indicated to help readers understand why these positions were picked and perhaps why one pair was better than the other. In both instances Fig 2d is cited as a basis for the selection of these positions but the figure does not indicate where these aa are. Alternatively, if the models of these structures also have the introduced disulfide modelled then the authors can show a magnified view of this region of the complex.

(3) Discussion. I wonder if there is scope for a little more speculation on the mechanism of action? The small size of the BepA pocket seems to indicate that only a completely unfolded polypeptide could enter BepA for digestion. How could fully embedded strands be unfolded and pulled out of the OM (especially given the energetic austerity of the OM)? Could any of the BAM rearrangements provide clues for this? Finally, it seems like BepA is positioned to specifically act on the N-termini of OMP barrels which might be worth stating (if the authors agree). Finally, BepA also appears to support on-pathway OMP folding. BepA chaperone function should be discussed – do any of the structures support the forward OMP folding pathway?

(4) Discussion: Statement lines 308-310 needs revisising as it is incorrect. Several papers of BAM-OMP stalled structures were cited but the authors then said that all of these structures have an unfolded strand just below the membrane that is unresolved. This is incorrect. Which strands are the authors referring to? At least for a few of the intermediates solved by Shen et al, Nature 2023 all OMP strands are accounted for in the map density.

Misc
1) Intro: Sentence starting line 47 needs citations.

2) Some coloured font too hard to see (light pink BepA and light turquoise LptD).

Reviewer #2

(Remarks to the Author)
Fenn et al. describe the cryoEM structures of BAM-BepA complexes, giving insights on how BepA degrades nascent outer membrane proteins stalled on Bam. Single particle analyses of in vitro reconstituted complexes show two major different conformations named the encounter and poised complexes. In the encounter complex Bam and BepA interact without rearrangements of the different components, while large conformational changes are observed in the poised complex, allowing the BepA catalytic site to be positioned close to the membrane and the BamA barrel where the folding of OMPs occurs. Despite the low resolution of the cryoEM maps, alphafold AF predictions and/or published structures of Bam components and BepA were clearly identified and fitted into the maps. Structural analysis of two additional complexes in which engineered cysteines were incorporated provided higher resolution structures, validating the results. In one of the maps the lid domain of BepA was clearly visible in the membrane and found to interact with the barrel of BamA. For all these structures the plug domain of BepA is in the closed conformation, with the His246 coordinated with a Zn ion. To understand how the plug domain opens with the dissociation of His246 from Zn, the authors used AF3 structure predictions of BepA in the presence of different peptides. Predictions in which the His246 was found not to interact with Zn were used to design peptides that were shown experimentally to be degraded by BepA.

Bam is an essential component of Gram negative bacteria and a potential target for the development of new antibiotics. The findings presented by Fenn et al. will be of interest to the audience of Nature Communications. However, the part that uses AF3 to analyze the movement of the plug of BepA is not convincing in the current form and needs further explanation and discussion.

One of the concerns with the AF3 prediction approach is the focus on BepA alone. Both the AF3 predictions and the peptide cleavage experiments are performed with BepA in solution. Do the authors assume that BepA has the same activity on its own versus bound to the Bam complex? Could the network of interaction between Bam and BepA regulate the proteolysis activity of BepA? This is an important aspect for the Bam-BepA activity that should be discussed in more details.
The use of AF3 to predict movement of a residue that is part of an active site is new to me. Has this approach been used anywhere else?
The methodology used with AF3 should be described more extensively. On the AF3 server, it is stated that pTM calculations tend to yield low values for chains shorter than 16 residues. How was this considered for the predictions of the 350 peptides tested? For the peptides that showed a displacement of His246 from Zn, were they all found to interact with the edge strand of BepA? And if so, did they have the same orientation? What is the expected orientation of the peptides in the BepA-Bam complex? Line 231-232, it is stated that “the zinc ion was no longer coordinated to the plug histidine but was instead replaced by a water molecule that would be required for catalysis”. AF3 is presumably not able to predict water molecules.

The addition of new figures would improve the paper. A schematic model added to figure 1 on how Bam folds OMPs and how BepA assist in the maturation process would help readers who are not familiar with the subjext. Another figure showing the structure of Bam bound to a substrate, like PDB 7TTC, together with the poised or lid-engaged structure would help to visualize the proximity and orientation of the BepA active site with a potential substrate.

Other comments:
Figures 4 and 5 could be one figure. Subpanels 4-e, 4-h and 4-I could be moved to supplementary information. 5-b is redundant to 5-a.
Supplementary figure 3: subpanels c and f should be removed. Hydrogen bonds and salt bridges cannot be inferred from low resolution structures using rigid body fits.
Line 148: “…the structures was worsened to >5Å but the b-factor remained unchanged”. What is the significance of the b-factor in this context?
Line 211: “…all Zn2+ coordination sites are full with protein ligands”. Please rephrase

Version 1:

Reviewer comments:

Reviewer #1

(Remarks to the Author)
The authors have fully addressed all of my concerns / comments. I look forward to seeing this manuscript published by Nature Communications.

Reviewer #2

(Remarks to the Author)
The authors have addressed most of my concerns. However, supplementary figure 3-c and f still indicate hydrogen bonds and salt bridges even though the authors claimed they removed the designation of bond types in their response to reviewers. I strongly suggest to remove hydrogen bond and salt bridges designation and replace them with close proximity for example.

Open Access This Peer Review File is licensed under a Creative Commons Attribution 4.0 International License, which permits use, sharing, adaptation, distribution and reproduction in any medium or format, as long as you give appropriate credit to the original author(s) and the source, provide a link to the Creative Commons license, and indicate if changes were made.
In cases where reviewers are anonymous, credit should be given to 'Anonymous Referee' and the source.
The images or other third party material in this Peer Review File are included in the article's Creative Commons license, unless indicated otherwise in a credit line to the material. If material is not included in the article's Creative Commons license and your intended use is not permitted by statutory regulation or exceeds the permitted use, you will need to obtain permission directly from the copyright holder.
To view a copy of this license, visit https://creativecommons.org/licenses/by/4.0/

Response to Reviewers

Reviewer #1 (Remarks to the Author):

Protein degradation at the bacterial outer membrane remains very poorly understood but one of the key proteases that have emerged in recent years is BepA. The importance of BepA is clear given the antibiotic permeability defects observed for a bepA null mutant. Here Fenn et al use cryoEM, AF3 predictions, and proteolysis assays to finely characterise previously unknown states of BepA as it is bound to BAM revealing a plausible path of conformational changes to bring the BepA active pocket in close proximity to where a new OMP would be folding into the membrane while bound to BamA in a hybrid-barrel conformation. This is excellent work and I would encourage publication in Nature Communications. I have only one minor comment potentially worth addressing with an additional experiment (1) and several other minor comments:

We thank the reviewer for their positive and encouraging comments.

(1) In Figure 2c the lid shut vancomycin sensitivity phenotype is interesting but the result would be much more meaningful if the level of disulfide formation was also determined. Is the phenotype resultant of 90% of BepA forming a disulfide between A64C-A173C or did only a small fraction of BepA oxidise? It might be nice to blot lysates to detect a BepA band shift that would correspond with the level of disulfide oxidation. Also, it would be helpful to readers to show on a model placed into the figure where the cysteines were engineered.

The purified Lid-shut BepA protein is fully oxidised (and the disulphide cannot be reduced using DTT). To show that the disulphide is fully formed we reacted the protein with I-AEDANS, using a BepA derivative with a single Cys as a positive control. The results show that Lid-Shut BepA does not label with I-AEDANS, consistent with full oxidation. Please see new Supplementary Figure 6 with this result and the position of the disulphide highlighted. We now refer to this in the Figure legend of Figure 2 and reference the figure in line 165.

(2) To trap the lid engaged complex the authors engineered intermolecular disulfides: BepA(R155C)-BamA(D211C) [moderate success], and BepA(L186C)-BamA(P723C) [high success]. It would be very helpful to add a model to the figure with these positions indicated to help readers understand why these positions were picked and perhaps why one pair was better than the other. In both instances Fig 2d is cited as a basis for the selection of these positions but the figure does not indicate where these aa are. Alternatively, if the models of these structures also have the introduced disulfide modelled then the authors can show a magnified view of this region of the complex.

We now include a new panel (Supplementary Data Figure 5k) to highlight the position of the engineered disulphides and refer to it accordingly in the text. We also include a new panel (Supplementary Figure 8k) to show the density for the disulphide in the lid-engaged Complex. The disulphide in the poised complex was unresolved.

(3) Discussion. I wonder if there is scope for a little more speculation on the mechanism of action? The small size of the BepA pocket seems to indicate that only a completely unfolded polypeptide could enter BepA for digestion. How could fully embedded strands be unfolded and pulled out of the OM (especially given the energetic austerity of the OM)? Could any of the BAM rearrangements provide clues for this? Finally, it seems like BepA is positioned to specifically act on the N-termini of OMP barrels which might be worth stating (if the authors agree). Finally, BepA also appears to support on-pathway OMP folding. BepA chaperone function should be discussed – do any of the structures support the forward OMP folding pathway?

These are all great questions. We have now expanded our discussion. Lines 316-324 address some of these questions.

How (and whether) fully embedded strands are pulled out of the OM is a fascinating question, but BAM rearrangements have not provided us with any clues. We prefer not to speculate further on this, other than raising it as an unanswered question and making the suggestions that perhaps Skp plays a role after BepA cleavage given its already established role doing this (lines 364-365) or that the lid might interact with OMP strands embedded in the membrane and facilitate their removal (lines 366-368).

Regarding the region so OMPs cleaved by BepA – this is another unanswered question for the future, but we assume that any region of the chain that has not yet folded into the OM and can access the BepA active site could potentially be a substrate.

Finally, regarding BepA chaperone activity ("on-pathway OMP folding"). We note that direct experimental evidence for a *bona fide* chaperone activity of BepA remains limited. While several studies suggest BepA promotes productive LptD biogenesis (ref 28, 31, 39) these observations are largely indirect based on genetic separation-of-function phenotypes and *in vivo* studies of LptD assembly intermediates with BepA mutants that are uncharacterised biochemically. We think these are hard to interpret as any changes to BepA will likely alter the sigma E response and lead to changes in the relative levels of other folding factors such as SurA, the BAM complex subunits as well as other proteases. These studies can be accommodated by models in which BepA functions in substrate surveillance or quality control during OMP assembly, rather than directly acting as a chaperone. As this activity is not considered in our experiments, which rather specifically ask how BepA degrades partially folding OMPs, we prefer not to discuss the chaperoning ability of BepA in our manuscript.

(4) Discussion: Statement lines 308-310 needs revising as it is incorrect. Several papers of BAM-OMP stalled structures were cited but the authors then said that all of these structures have an unfolded strand just below the membrane that is unresolved. This is incorrect. Which strands are the authors referring to? At least for a few of the intermediates solved by Shen et al, Nature 2023 all OMP strands are accounted for in the map density.

We agree the statement was misleading and we have amended it. However, all the structures the referee refers to do have regions of unresolved polypeptide chain leading to tags or unresolved yet-to-fold OMP strands that are present but unresolved in the EM maps. This includes the cited paper from Shen *et al*, in which the constructs all have regions of the EspP linker connecting to the MBP tag that are unresolved in their structures (see Figure 5 of our recent review Birtles et al 2026). The sentence has now been changed to read: *'In each of these, BepA's active site would sit below the nascent OMP barrels, ideally placed to cleave the unfolded OMP chain just below the membrane (all BAM-OMP stalled structures contain regions of unresolved polypeptide chain).'*

Misc
1) Intro: Sentence starting line 47 needs citations.
We have added relevant references.

2) Some coloured font too hard to see (light pink BepA and light turquoise LptD).
We were unsure exactly which figure this refers to but have endeavoured to update the colours of labels including the LptD label in Figure 4 and hope this has resolved any issues.

Reviewer #2 (Remarks to the Author):

Fenn et al. describe the cryoEM structures of BAM-BepA complexes, giving insights on how

BepA degrades nascent outer membrane proteins stalled on Bam. Single particle analyses of in vitro reconstituted complexes show two major different conformations named the encounter and poised complexes. In the encounter complex Bam and BepA interact without rearrangements of the different components, while large conformational changes are observed in the poised complex, allowing the BepA catalytic site to be positioned close to the membrane and the BamA barrel where the folding of OMPs occurs. Despite the low resolution of the cryoEM maps, alphafold AF predictions and/or published structures of Bam components and BepA were clearly identified and fitted into the maps. Structural analysis of two additional complexes in which engineered cysteines were incorporated provided higher resolution structures, validating the results. In one of the maps the lid domain of BepA was clearly visible in the membrane and found to interact with the barrel of BamA. For all these structures the plug domain of BepA is in the closed conformation, with the His246 coordinated with a Zn ion. To understand how the plug domain opens with the dissociation of His246 from Zn, the authors used AF3 structure predictions of BepA in the presence of different peptides. Predictions in which the His246 was found not to interact with Zn were used to design peptides that were shown experimentally to be degraded by BepA.

Bam is an essential component of Gram negative bacteria and a potential target for the development of new antibiotics. The findings presented by Fenn et al. will be of interest to the audience of Nature Communications. However, the part that uses AF3 to analyze the movement of the plug of BepA is not convincing in the current form and needs further explanation and discussion.

We thank the reviewer for the positive evaluation of our work and have carefully addressed their concerns as detailed below.

One of the concerns with the AF3 prediction approach is the focus on BepA alone. Both the AF3 predictions and the peptide cleavage experiments are performed with BepA in solution. Do the authors assume that BepA has the same activity on its own versus bound to the Bam complex? Could the network of interaction between Bam and BepA regulate the proteolysis activity of BepA? This is an important aspect for the Bam-BepA activity that should be discussed in more details.

We agree that the absence of BAM from our peptide cleavage experiments is a limitation of our study. The AF3 predictions with OMP peptides were completed in the absence of BAM to remove the possibility of the peptides interacting with BAM. (For example, if we use Alphafold to model a β-signal peptide in the presence of BAM it will, as might be expected, interact with BamA β-1.)

That BepA is functional in the absence of BAM is perhaps surprising, but this finding does confirm our hypothesis that the substrate, not BAM, moves the plug. To address this reviewer's comment, we now include results showing the proteolysis of the QMN peptide by BepA in the presence of BAM. The results show there is no visible difference in the rate of cleavage of this peptide in the presence of BAM (new Supplementary Figure 10). However, this experiment is limited as BAM is in detergent and we have not determined the affinity of BepA for BAM in DDM. However, we assume it is low affinity given our difficulties assembling the complexes by cryoEM. Therefore, we added BAM at the highest feasible excess within the constraints of our experiment (42 μM, 14x molar excess cf. BepA), so that even a low percent bound would give a measurable increase in degradation should binding enhance BepA catalytic activity. We would also point out that this does not mean BAM does not regulate BepA's activity, and point out that our experiment is not close to conditions that BepA likely encounters BAM and substrate in an *in vivo* context (i.e. BAM is not stalled with an OMP, it is not in a native membrane and our experiment is with a 15-residue peptide, not an OMP chain). We have added a sentence to our discussion about the limitations of this experiment and that future

work should focus on BepA's activity whilst bound to BAM with longer polypeptide segments from OMPs and in a more realistic membrane environment (see lines 319-323).

The use of AF3 to predict movement of a residue that is part of an active site is new to me. Has this approach been used anywhere else?

The reviewer is correct: the use of AlphaFold3 to infer movement of active-site residues or conformational rearrangements is an emerging application of the method. A search of the literature shows there are other examples (the original AlphaFold3 paper includes some examples (Abramson et al 2024, our ref 32)).

Gao et al 2022 (ref 34) also used AlphaFold2 to predict complexes of ~1500 "super protein complexes for outer membrane protein biogenesis in bacteria". In this work the authors suggest possible conformational rearrangements of many proteins within the context of the OM. They also predict LepB, the signal peptidase, in the presence of proOmpA chain to suggest how LepB accesses a substrate to cleave signal peptide.

A recent BioRxiv paper (Tang et al 2026, Prediction of ligand-dependent conformational sampling of ABC transporters by AlphaFold3 and correlation to experimental structures and energetics (doi: https://doi.org/10.64898/2026.02.19.706864)) correlates AF3 predictions with experimental structures when ABC transporters bind ligands and conformationally change. They conclude that AF3 provides a tool for studying how ligand binding shifts protein conformation equilibrium.

Finally, another recent BioRxiv paper (Lazou et al 2026, The influence of ligands on AlphaFold3 prediction of cryptic pockets (doi: https://doi.org/10.64898/2026.01.04.697564)) studies the use of AF3 for generating realistic conformational ensembles that reveal cryptic pockets. AF3 was found to be able to generate open-pocket conformations that matched experimentally observed ligand-bound states.

The methodology used with AF3 should be described more extensively. On the AF3 server, it is stated that pTM calculations tend to yield low values for chains shorter than 16 residues. How was this considered for the predictions of the 350 peptides tested?

We note that pTM is primarily intended as a global fold confidence metric and is not optimally suited for evaluating peptide-protein interaction models, particularly for short peptides where the score is known to be unreliable. In our study, therefore, we did not interpret pTM as a measure of interaction validity. Instead, we assessed convergence of peptide binding poses across multiple independent AF3 runs as a proxy for interaction robustness. For this reason in Figure 4e we only designate a particular peptide as moving the plug when ≥4 of 5 models move the plug, or conversely to have no effect when ≥4 of 5 models do not move the plug.

For the peptides that showed a displacement of His246 from Zn, were they all found to interact with the edge strand of BepA? And if so, did they have the same orientation? What is the expected orientation of the peptides in the BepA-Bam complex?

We have now analysed all of the peptides that moved the plug for their interaction with the edge strand. This analysis showed that 96% of these peptides indeed interact with the edge β-strand (where an interaction was defined as any carbon-α in the peptide within 5Å of any carbon-α in the BepA edge β strand (residues 105-100)). 25% of peptides formed β-strands as defined by DSSP (which weights h-bond formation as the metric) and 85% of which were anti-parallel. We have added a sentence at line 246-249 to describe this. It has not been experimentally validated in the literature if OMPs do template against the edge strand, nor

their expected orientation known if they do. These data have also been deposited in the University of Leeds doi.

Line 231-232, it is stated that "the zinc ion was no longer coordinated to the plug histidine but was instead replaced by a water molecule that would be required for catalysis". AF3 is presumably not able to predict water molecules.

AF3 can predict ligands, a capability that wasn't available in AF2. The AlphaFold server cannot predict water molecules, but AF3 installed locally can. Figure 4d shows the coordination of zinc by a water molecule as predicted by AF3.

The addition of new figures would improve the paper. A schematic model added to figure 1 on how Bam folds OMPs and how BepA assist in the maturation process would help readers who are not familiar with the subjext. Another figure showing the structure of Bam bound to a substrate, like PDB 7TTC, together with the poised or lid-engaged structure would help to visualize the proximity and orientation of the BepA active site with a potential substrate.

Thank you for this suggestion. We now direct the reader to our recent review in Chem Reviews (Birtles et al 2026) for an overview of the BAM complex and how it folds OMPs (which has been well described before and is not the focus of our manuscript). However, we have now included a new supplementary figure to demonstrate how the active site of BepA is positioned close to the site on BAM where folding of OMP into the OM occurs (new Supplementary Data Figure 12).

Other comments:
Figures 4 and 5 could be one figure. Subpanels 4-e, 4-h and 4-I could be moved to supplementary information. 5-b is redundant to 5-a.

Supplementary figure 3: subpanels c and f should be removed. Hydrogen bonds and salt bridges cannot be inferred from low resolution structures using rigid body fits.

Thank you for your suggestion. Supplementary Figure 3f shows the interacting residues for the Poised complex that was model built in our map of the disulphide bond Poised complex (resolution 3.9Å) and then rigid body docked into our low resolution map. We agree that we cannot infer the hydrogen bonds in Supplementary Figure 3c from a low resolution structure but keep the table as we think they may be potentially useful to a reader who might like to know roughly what residues are interacting but remove the designation of bond type and softened the language in our legend to show that we can't be confident in these interactions.

Line 148: "…the structures was worsened to >5Å but the b-factor remained unchanged". What is the significance of the b-factor in this context?

In cryoEM, the B-factor describes the attenuation of the signal at high frequencies, essentially measuring how the map's sharpness or signal-to-noise ratio decreases relative to spatial resolution. It serves as a measure of data degradation caused by optical imperfections, noise-induced errors in particle alignment, and molecular flexibility. Cryo-EM maps are often "blurred" in the final reconstruction and a negative B-factor is applied during post-processing to boost high-frequency signal and "sharpen" the map, enhancing details. In these structures the overall resolution decreased because alignment was effectively focused on the small more flexible BepA rather than the larger BAM complex. However, the unchanged b-factor did not worsen suggesting that the underlying high-frequency signal remained comparable.

Line 211: "…all Zn2+ coordination sites are full with protein ligands". Please rephrase

We have rephrased this to read "all Zn^{2+} coordination sites are occupied by protein ligands".

REVIEWERS' COMMENTS

Reviewer #1 (Remarks to the Author):

The authors have fully addressed all of my concerns / comments. I look forward to seeing this manuscript published by Nature Communications.

We thank the reviewer for their positive response.

Reviewer #2 (Remarks to the Author):

The authors have addressed most of my concerns. However, supplementary figure 3-c and f still indicate hydrogen bonds and salt bridges even though the authors claimed they removed the designation of bond types in their response to reviewers. I strongly suggest to remove hydrogen bond and salt bridges designation and replace them with close proximity for example.

We thank the reviewer for their comments and have removed the designation of bonding type, amending the legend appropriately.
