# Supplementary material for: BAM-BepA complexes in outer membrane protein quality control
Source: Nat Commun. 2026 Jul 9;17:8482. doi: 10.1038/s41467-026-75227-x (PMC13478230; doi:10.1038/s41467-026-75227-x)
Supplement: Supplementary file 5 [file 41467_2026_75227_MOESM7_ESM.zip › SourceData/Uncropped gels and plates.docx]

Fig2c-Vancomycin_200ugmL_BepA_deltaBepA_ΔNterx3

Fig2c-Vancomycin_200ugmL_BepA_ΔBepA_LidShutx3

Supplementary Figure 6 uncropped gels
